# Erythropoietin receptor signal is crucial for periodontal ligament stem cell-based tissue reconstruction in periodontal disease

**DOI:** 10.1038/s41598-024-57361-y

**Published:** 2024-03-20

**Authors:** MHD. Fouad Zakaria, Soichiro Sonoda, Hiroki Kato, Lan Ma, Norihisa Uehara, Yukari Kyumoto-Nakamura, M. Majd Sharifa, Liting Yu, Lisha Dai, Erika Yamauchi-Tomoda, Reona Aijima, Haruyoshi Yamaza, Fusanori Nishimura, Takayoshi Yamaza

**Affiliations:** 1 Department of Molecular Cell Biology and Oral Anatomy, Kyushu University Graduate School of Dental Science, 3-1-1 Maidashi, Higashi-ku, Fukuoka, 812-8582 Japan; 2 Department of Periodontology, Kyushu University Graduate School of Dental Science, Fukuoka, Japan; 3 Guangdong Provincial Key Laboratory of Stomatology, South China Center of Craniofacial Stem Cell Research, Hospital of Stomatology, Guanghua School of Stomatology, Sun Yat-sen University, Guangzhou, China; 4 Department of Oral and Maxillofacial Radiology, Kyushu University Graduate School of Dental Science, Fukuoka, Japan; 5 Department of Oral and Maxillofacial Surgery, Faculty of Medicine, Saga University, Saga, Japan; 6 Department of Pediatric Dentistry, Kyushu University Graduate School of Dental Science, Fukuoka, Japan

**Keywords:** Chronic inflammation, Periodontitis, Mesenchymal stem cells

## Abstract

Alveolar bone loss caused by periodontal disease eventually leads to tooth loss. Periodontal ligament stem cells (PDLSCs) are the tissue-specific cells for maintaining and repairing the periodontal ligament, cementum, and alveolar bone. Here, we investigated the role of erythropoietin receptor (EPOR), which regulates the microenvironment-modulating function of mesenchymal stem cells, in PDLSC-based periodontal therapy. We isolated PDLSCs from patients with chronic periodontal disease and healthy donors, referred to as PD-PDLSCs and Cont-PDLSCs, respectively. PD-PDLSCs exhibited reduced potency of periodontal tissue regeneration and lower expression of EPOR compared to Cont-PDLSCs. *EPOR*-silencing suppressed the potency of Cont-PDLSCs mimicking PD-PDLSCs, whereas EPO-mediated EPOR activation rejuvenated the reduced potency of PD-PDLSCs. Furthermore, we locally transplanted *EPOR*-silenced and EPOR-activated PDLSCs into the gingiva around the teeth of ligament-induced periodontitis model mice and demonstrated that EPOR in PDLSCs participated in the regeneration of the periodontal ligament, cementum, and alveolar bone in the ligated teeth. The EPOR-mediated paracrine function of PDLSCs maintains periodontal immune suppression and bone metabolic balance via osteoclasts and osteoblasts in the periodontitis model mice. Taken together, these results suggest that EPOR signaling is crucial for PDLSC-based periodontal regeneration and paves the way for the development of novel options for periodontal therapy.

## Introduction

Periodontitis is one of the most common chronic inflammatory diseases in humans, and 15% of patients are diagnosed with severe periodontitis^1,2^. Periodontitis is initiated by oral bacterial dysbiosis colonizing on subgingival tooth surfaces^3,4^. The dysbiosis eventually results in inflammatory destruction of bone and connective tissues in the periodontium and tooth loss^5^. Gold-standard treatment with scaling and root planing aims to treat periodontitis and restore periodontal health. Current periodontal regenerative surgery restores, at least partially, periodontal pockets in the damaged periodontium. However, the post-operative regeneration of the periodontal bone and tissue defects remains hard due to the less innate repairing ability of damaged periodontium^6^. Thus, developing clinically effective options is argued to acquire enough healthy periodontal tissues to restore the architecture and function of periodontium^6,7^.

The periodontal ligament (PDL), a dense fibrous connective tissue connecting the tooth root to the surrounding alveolar bone, contains a niche for tissue-specific stem cells, namely PDL stem cells (PDLSCs)^8^. PDLSCs exhibit in vitro and in vivo potency in PDL cells and cementoblasts/cementocytes and contribute to the homeostasis, repair, and regeneration of PDL and cementum. PDLSCs also exert an immunosuppressive effect on immune cells^9^, suggesting that they actively function in microenvironmental modulation and tissue homeostasis in the PDL, cementum, and alveolar bone.

Erythropoietin receptor (EPOR), a member of the type I cytokine receptor family, is expressed on the surface of erythroid progenitors^10^. Its ligand, erythropoietin (EPO), is an N-linked glycoprotein hormone secreted by the kidney that attacks erythroid progenitors and eventually mediates EPOR/Janus kinase/signal transducer and activator of transcription 5 (STAT5) pathway to hematopoiesis in the bone marrow^11^. EPO induces the erythroid transcription factors GATA-binding protein 1 and TAL BHLH transcription factor 1, leading to the epigenetic transactivation of EPOR^12,13^. Deletion of *EPOR* in mice causes embryonic death owing to severe anemia^14^, whereas the erythroid-targeting knock-in of *EPOR* rescues the life of *EPOR* knock-out mice^15^. In contrast, EPO/EPOR signaling regulates metabolic responses in nonhematopoietic tissues, including the brain, heart, skeletal muscle, and adipose tissue^16,17^. A recent study evaluated the novel action of EPO/EPOR/STAT5 signaling in regulating the microenvironment-modulating function of bone marrow mesenchymal stem cells (BMMSCs)^18^. EPO treatment accelerated in vitro mineralized tissue formation in PDLSCs via several pathways, including p38 mitogen-activated protein kinase and Wnt/β-catenin^19,20^. EPO treatment also attenuated the impaired function of oxidative stress and mineralized tissue formation in PDLSCs under high glucose levels^21^. However, the role of the EPOR pathway in modulating the microenvironment of healthy and pathological PDLSCs remains unknown. We hypothesized that EPOR contributes to the periodontal regenerative potency of PDLSCs. Gene silencing and ligand activation of EPOR function determined whether EPOR signaling was involved in PDLSC functions in tissue formation of the PDL and cementum, immunosuppression of neutrophils, and alveolar bone turnover by osteoclasts and osteoblasts. Furthermore, a PDLSC transplant assay revealed that the EPOR pathway regulates the therapeutic efficacy of PDLSCs in periodontal tissue destruction and inflammation in a ligature-induced periodontitis model. Thus, in the present study, we test how EPOR pathways in PDLSCs contribute to recovering the tissue damage in the periodontium including PDL, cementum, and alveolar bone.

## Results

### Microenvironment-modulating dysfunctions of periodontitis-specific PDLSCs are associated with EPOR reduction

To evaluate our hypothesis, we investigated the mimicking of EPOR-knockdown control PDLSCs to periodontitis patient-derived PDLSCs (PD-PDLSCs) and the successful rejuvenation of the dysfunction of PD-PDLSCs via EPO-EPOR signaling. Therefore, in this study, we used young-donor-derived healthy PDLSCs, not the patient-age-matched donor-derived healthy PDLSCs, as the control healthy PDLSCs (Cont-PDLSCs) to the patient-derived PDLSCs.

PD-PDLSCs exhibited lower capabilities of colony formation and cell surface marker expression than Cont-PDLSCs (Fig. S1a–d). PD-PDLSCs showed the suppressed ability of population doubling level, telomerase activity, *telomerase reverse transcriptase* (*TERT*) expression, and cell proliferation by population doubling assay, telomerase repeat amplification protocol and quantitative polymerase chain reaction (TRAP-qPCR), reverse transcription-quantitative polymerase chain reaction (RT-qPCR), and bromodeoxyuridine (BrdU) incorporation assay (Fig. S1e–h). Cont-PDLSCs suppressed the in vitro cell survival of anti-CD3 epsilon (CD3e) antibody-activated human peripheral blood mononuclear cells (PBMNCs) but PD-PDLSCs not (Fig. S1i). PD-PDLSCs showed lower in vitro multipotency into adipocytes, chondrocytes, ligament cells, and cementoblasts than Cont-PDLSCs by specific matrix staining and specific gene expression analysis (Fig. S2a–h). When PDLSCs were subcutaneously transplanted into immunocompromised mice, Cont-PDLSCs exhibited superior in vivo periodontal tissue regeneration of a cementum-like mineralized matrix, dense and bundled-like fibers, and Sharpey’s fiber-like structures but PD-PDLSCs showed the less regenerative properties compared to Cont-PDLSCs by hematoxylin and eosin (H&E) staining and immunostaining for human mitochondria (Fig. S2i, j).

**Figure 1:**
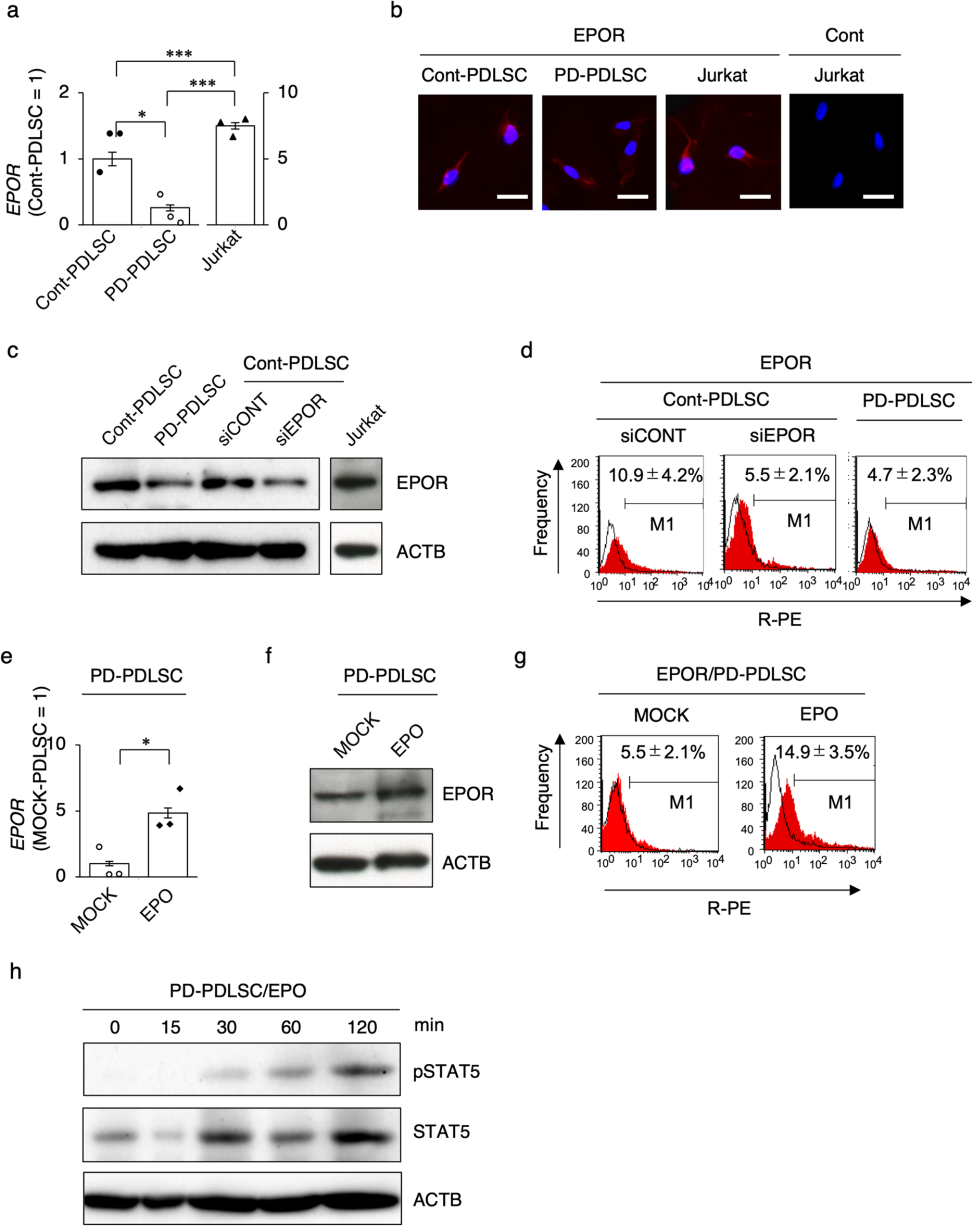
Expression of erythropoietin receptor in human periodontal ligament stem cells. (**a**) Expression of erythropoietin receptor (EPOR) in periodontal ligament stem cells (PDLSCs) revealed by reverse transcription-quantitative polymerase chain reaction (RT-qPCR). The results are shown as a ratio to the expression in healthy control PDLSCs (Cont-PDLSCs) (Cont-PDLSC = 1). (**b**) Representative immunofluorescent images of EPOR expression in PDLSCs. Cont, Cont-IgG antibody staining. (**c**) Representatives immunoblot images of expression of EPOR in PDLSCs. Original blots are presented in Figs. S6, S7. (**d**) Representative histograms of EPOR expression in PDLSCs by flow cytometry analysis. (**e–h**) PD-PDLSCs were treated with recombinant erythropoietin (EPO) and PBS (MOCK). Expression of EPOR in PD-PDLSCs by RT-qPCR. The results are shown as a ratio to the expression in MOCK treated PD-PDLSCs (MOCK-PDLSCs) (MOCK-PDLSC = 1) (**e**). Representative immunoblot images of EPOR expression in PD-PDLSCs. Original blots are presented in Fig. S7 (**f**). Representative histograms of EPOR expression in PD-PDLSCs by flow cytometry analysis(**g**). Representative immunoblot images of total signal transducer and activator of transcription 5 (STAT5) and phosphorylated STAT5 (pSTAT5) expression in PD-PDLSCs. Original blots are presented in Fig. S8 (**h**). (**a–h**) PD-PDLSC, periodontitis specific PDLSC. (**a–c**) Jurkat cells were used as a positive control for EPOR expression. (**a,e**) Data represent the mean ± SEM. *n* = 3/group. Significance was determined by independent two-tailed Student’s *t*-test; *P < 0.05. (**c,d**) siEPOR, siRNA for *EPOR* treated group; siCONT, scrambled control siRNA treated group. (**c,f,h**) ACTB, actin beta. (**d,g**) Positive rates (%) are presented as the mean ± SEM. *n* = 3/group. White area: histograms stained with control antibody; red area: histograms stained with antibodies against EPOR. *R-PE* R-phycoerythrin.

RT-qPCR analysis showed that both Cont-PDLSCs and PD-PDLSCs expressed *EPOR* but PD-PDLSCs exhibited lower expression of *EPOR* than Cont-PDLSCs (Fig. 1a). The *EPOR* expression was supported by the EPOR expression in both Cont-PDLSCs and PD-PDLSCs, determined by immunofluorescent and immunoblot analyses and flow cytometry (Fig. 1b–d). We then functionally downregulated EPOR in Cont-PDLSCs using small interfering RNA (siRNA) transfection. The effect of siRNA for EPOR (siEPOR) on Cont-PDLSCs (siEPOR-PDLSCs) displayed suppressed expression of EPOR compared to that of the control scrambled siRNA (siCONT) on Cont-PDLSCs (siCONT-PDLSCs) by immunoblot analysis and flow cytometry (Fig. 1c, d). Interestingly, PD-PDLSCs showed a similar expression level of EPOR to siEPOR-PDLSCs by immunoblot analysis and flow cytometry (Fig. 1c, d). We examined the effects of EPO on PD-PDLSCs. RT-qPCR revealed that EPO treatment enhanced the expression of the EPOR gene and protein in PD-PDLSCs by RT-qPCR, immunoblot analysis, and flow cytometry (Fig. 1e–g). EPO treatment induced phosphorylation of STAT5 in PD-PDLSCs (Fig. 1h). Given the effects of EPO/EPOR/STAT5 signaling on the microenvironment-modulating potency of BMMSCs^18^, the present findings suggested that EPO/EPOR signaling in PDLSCs may participate in recovering periodontal tissue damage.

### Reduced EPOR expression participates in impaired microenvironment-modulating function in PDLSCs

**Figure 2:**
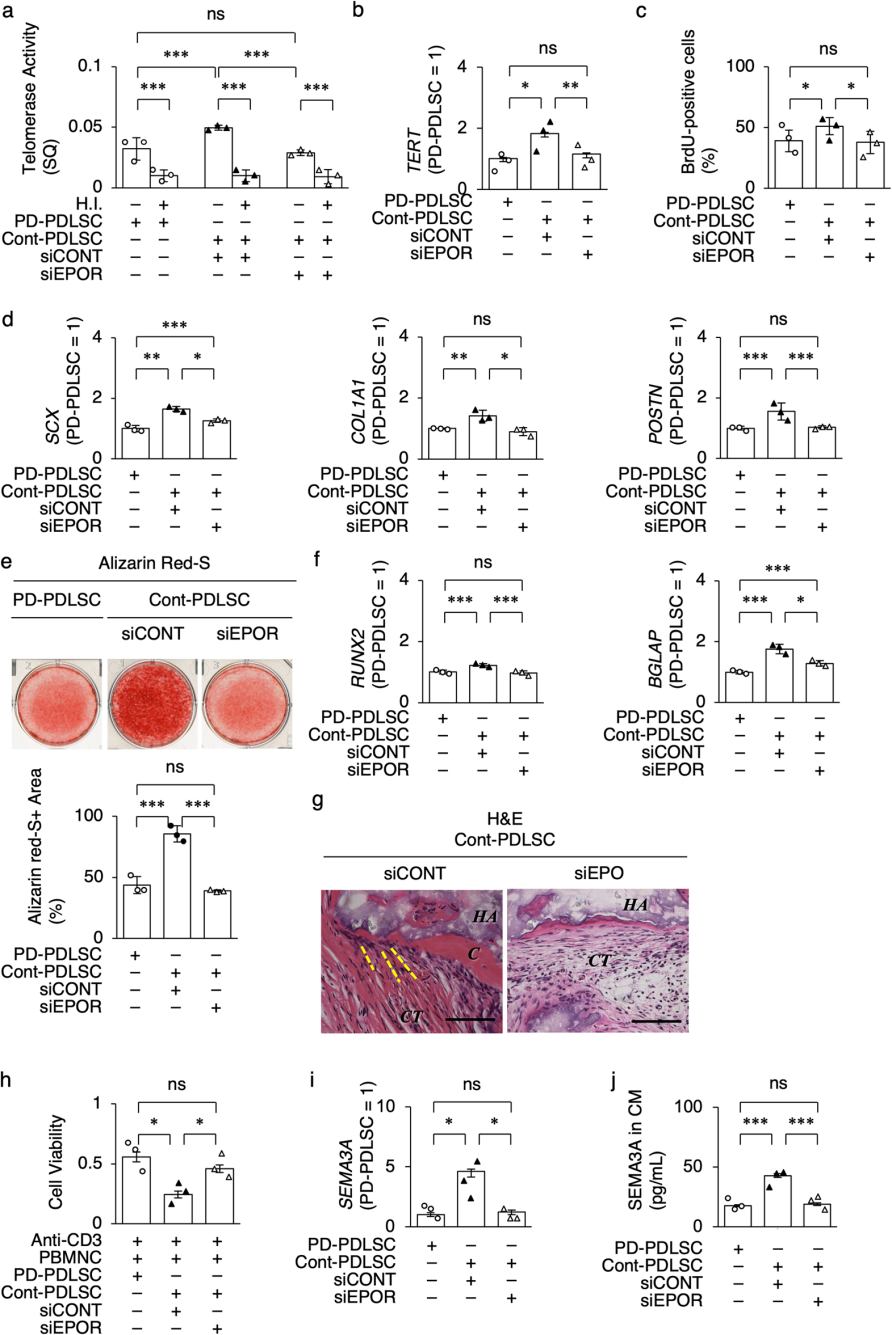
EPOR gene silencing impairs the periodontal tissue regenerative potency of human PDLSCs. (**a**) Telomerase activity in PDLSCs by telomerase repeat amplification protocol and qPCR (TRAP-qPCR). H.I., heat-inactivated samples. SQ, threshold cycles. (**b**) Expression of *telomerase reverse transcriptase* (*TERT*) in PDLSCs by RT-qPCR. (**c**) Cell proliferation capacity of PDLSCs by bromodeoxyuridine (BrdU) incorporation assay. (**d**) Expression of *scleraxis BHLH transcription factor* (*SCX*), *collagen type I alpha 1 chain* (*COL1A1*), and *periostin* (*POSTN*) in ligamentogenic PDLSCs by RT-qPCR. (**e**) Representative images of mineralized nodules of cementogenic/osteogenic PDLSCs by Alizarin Red-S staining. Ratio (%) of Alizarin Red-S-positive (Alizarin Red-S+) area in cementogenic/osteogenic PDLSCs. (**f**) Expression of *runt-related family transcription factor 2* (*RUNX2*) and *bone gamma-carboxyglutamate protein* (*BGLAP*) in cementogenic/osteogenic PDLSCs by RT-qPCR. (**g**) Representative histological images of subcutaneous implant tissues of PDLSCs by hematoxylin and eosin staining. *C*, cementum-like mineralized tissue; *CT*, fibrous connective tissue; *HA*, hydroxyapatite and tricalcium phosphate. Yellow dot line, Sharpey’s fiber-like structure. Scale bars, 100 mm. (**h**) Immunosuppressive function of PDLSCs to anti-CD3 epsilon (CD3e) antibody (Anti-CD3) activated human peripheral blood mononuclear cells (PBMNCs) by cell viability assay. (**I**) Expression of semaphoring 3A (*SEMA3A*) in PDLSCs by RT-qPCR. (**j**) Levels of SEMA3A in conditioned medium (CM) of PDLSCs by enzyme-linked immunosorbent assay (ELISA). (**a–f,h–j**) Data are presented as the mean ± SEM. *n* = 3/group. Significance was determined by two-way ANOVA with Tukey’s post hoc test; *P < 0.05, **P < 0.01, and ***P < 0.005. no significance (ns). (**b,d,f,i**) The results are shown as a ratio to the expression in PD-PDLSCs (PD-PDLSC = 1).

Next, we investigated whether siEPOR-PDLSCs exhibit less microenvironment function compared to siCONT-PDLSCs. siEPOR-PDLSCs exhibited suppressed cellular functions of telomerase activity, *TERT* expression, and cell proliferation capacity compared with siCONT-PDLSCs by TRAP-qPCR, RT-qPCR, and BrdU incorporation assay (Fig. 2a–c). RT-qPCR evaluated that siEPOR-PDLSCs showed the reduced ligamentogenic gene expression of scleraxis BHLH transcription factor* I* (*SCX*), *collagen type I alpha 1 chain* (*COL1A1*), and *periostin* (*POSTN*) compared to siCONT-PDLSCs(Fig. 2d). siEPOR-PDLSCs also exhibited impaired calcified nodule formation and suppressed cementogenic/osteogenic gene expression of runt-related transcription factor 2 (*RUNX2*) and bone gamma-carboxyglutamate protein (*BGLAP*) compared to siCONT-PDLSCs by Alizarin Red S staining and RT-qPCR (Fig. 2e, f). Histological analysis demonstrated that siEPOR-PDLSCs exhibited reduced in vivo formation of cementum-like tissue, PDL-like dense ligaments, and Sharpey’s fiber-like structures compared with siCONT-PDLSCs (Fig. 2G). siEPOR-PDLSCs showed a decreased in vitro immunosuppressive function of anti-CD3e antibody-activated PBMNCs compared to siCONT-PDLSCs (Fig. 2h). Semaphorin 3A (SEMA3A) acts as an osteoprotective factor secreted from osteoblasts^22^ and participates in stemness and differentiation of PDLSCs^23^. SEMA3A is also involved in suppressing T-cell proliferation^24^. siEPOR-PDLSCs suppressed the gene expression and secretion of SEMA3A compared to siCONT-PDLSCs by RT-qPCR and enzyme-linked immunosorbent assay (ELISA) (Fig. 2i, j). These findings suggested that EPOR signaling plays a crucial role in the microenvironment-modulating function of PDLSCs.

### EPO-induced EPOR signal rejuvenates dysfunctions of PD-PDLSCs

**Figure 3:**
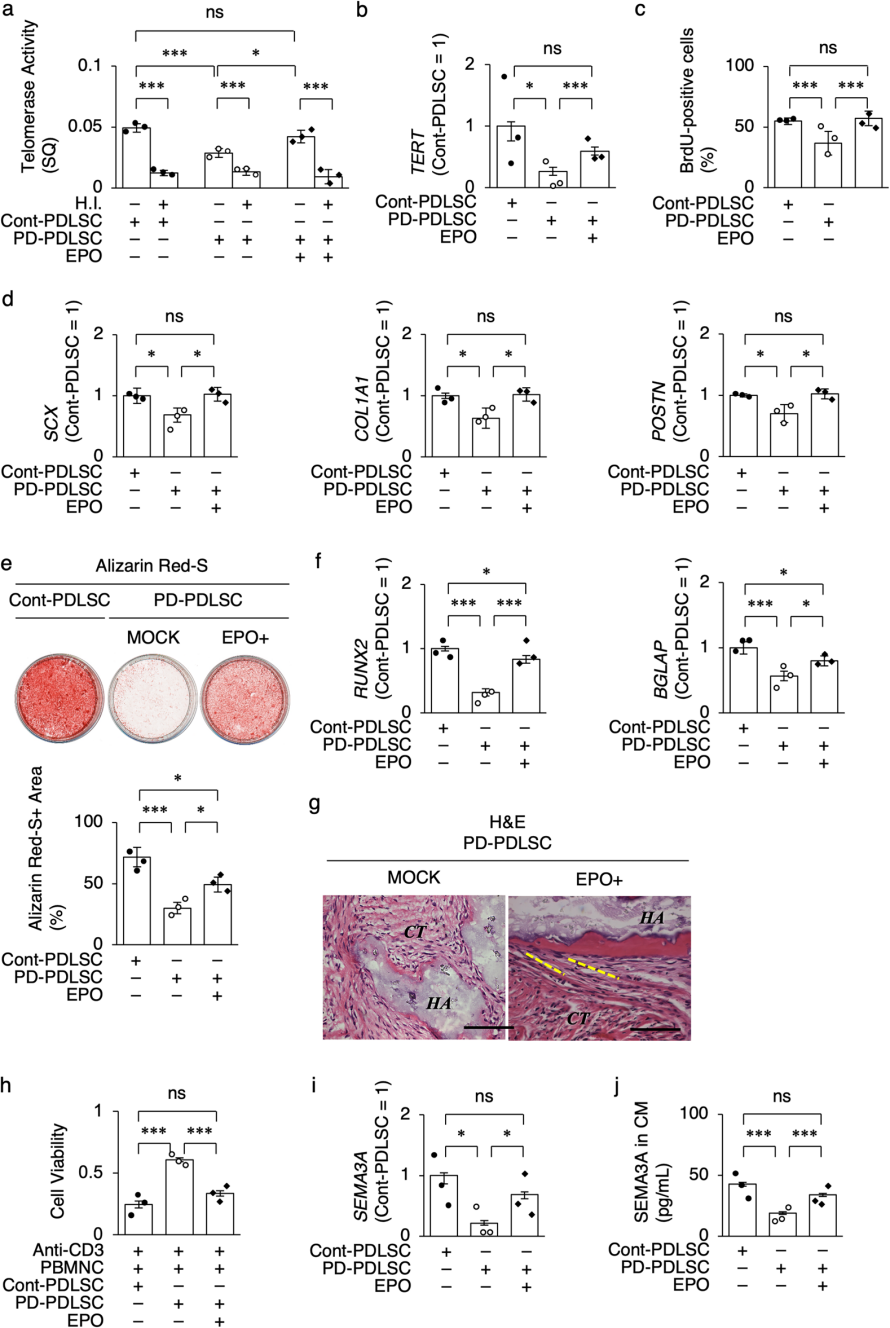
EPO treatment restores periodontal tissue regenerative potency of disease-specific PD-PDLSCs. (**a**) Telomerase activity in PDLSCs by TRAP-qPCR. H.I., heat-inactivated samples. SQ, threshold cycles. (**b**) Expression of *TERT* in PDLSCs by RT-qPCR. (**c**) Cell proliferation capacity of PDLSCs by BrdU incorporation assay. (**d**) Expression of *SCX*, *COL1A1*, and *POSTN* in ligamentogenic PDLSCs by RT-qPCR. (**e**) Representative images of mineralized nodules of cementogenic/osteogenic PDLSCs by Alizarin Red-S staining. Ratio (%) of Alizarin Red-S-positive area of cementogenic/osteogenic PDLSCs. (**f**) Expression of *RUNX2* and *BGLAP* in cementogenic/osteogenic PDLSCs by RT-qPCR. (**g**) Representative histological images of subcutaneous implant tissues of PDLSCs by hematoxylin and eosin (H&E) staining. *C*, cementum-like mineralized tissue; *CT*, fibrous connective tissue; *HA*, hydroxyapatite and tricalcium phosphate. Yellow dot line, Sharpey’s fiber-like structure. Scale bars, 100 mm. (**h**) Immunosuppressive function of PDLSCs to anti-CD3e antibody (Anti-CD3) activated PBMNCs by cell viability assay. (**i**) Expression of *SEMA3A* in PDLSCs by RT-qPCR. (**j**) Levels of SEMA3A in CM of PDLSCs by ELISA. (**a–e,h–j**) Data are presented as the mean ± SEM. *n* = 3/group. Significance was determined by two-way ANOVA with Tukey’s post hoc test; *P < 0.05 and ***P < 0.005. no significance (ns). (**b,d,f,i**) The results are shown as a ratio to the expression in Cont-PDLSCs (Cont-PDLSC = 1).

EPO-treated PD-PDLSCs (EPO-PD-PDLSCs) rejuvenated telomerase activity, *TERT* expression, and cell proliferation compared to MOCK (PBS) -treated PD-PDLSCs (MOCK-PD-PDLSCs) (Fig. 3a–c) and restored the decreased in vitro capacity of PD-PDLSCs to differentiate into ligament cells and cementoblasts/osteoblasts by RT-qPCR and Alizarin Red S staining (Fig. 3d–f). EPO-PD-PDLSCs recovered the in vivo periodontal tissue formation of cementum-like tissue, PDL-like dense ligaments, and Sharpey’s fiber-like structures compared to MOCK-PD-PDLSCs (Fig. 3g). EPO-PD-PDLSCs restored the in vitro immunosuppressive function of anti-CD3e antibody-activated PBMNCs and the in vitro SEMA3A production compared to MOCK-PD-PDLSCs (Fig. 3h–j), indicating that EPO effectively rejuvenate the microenvironment-modulating function of PD-PDLSCs.

### EPOR signal-regulated paracrine activity governs the microenvironment-modulating functions of PD-PDLSCs

**Figure 4:**
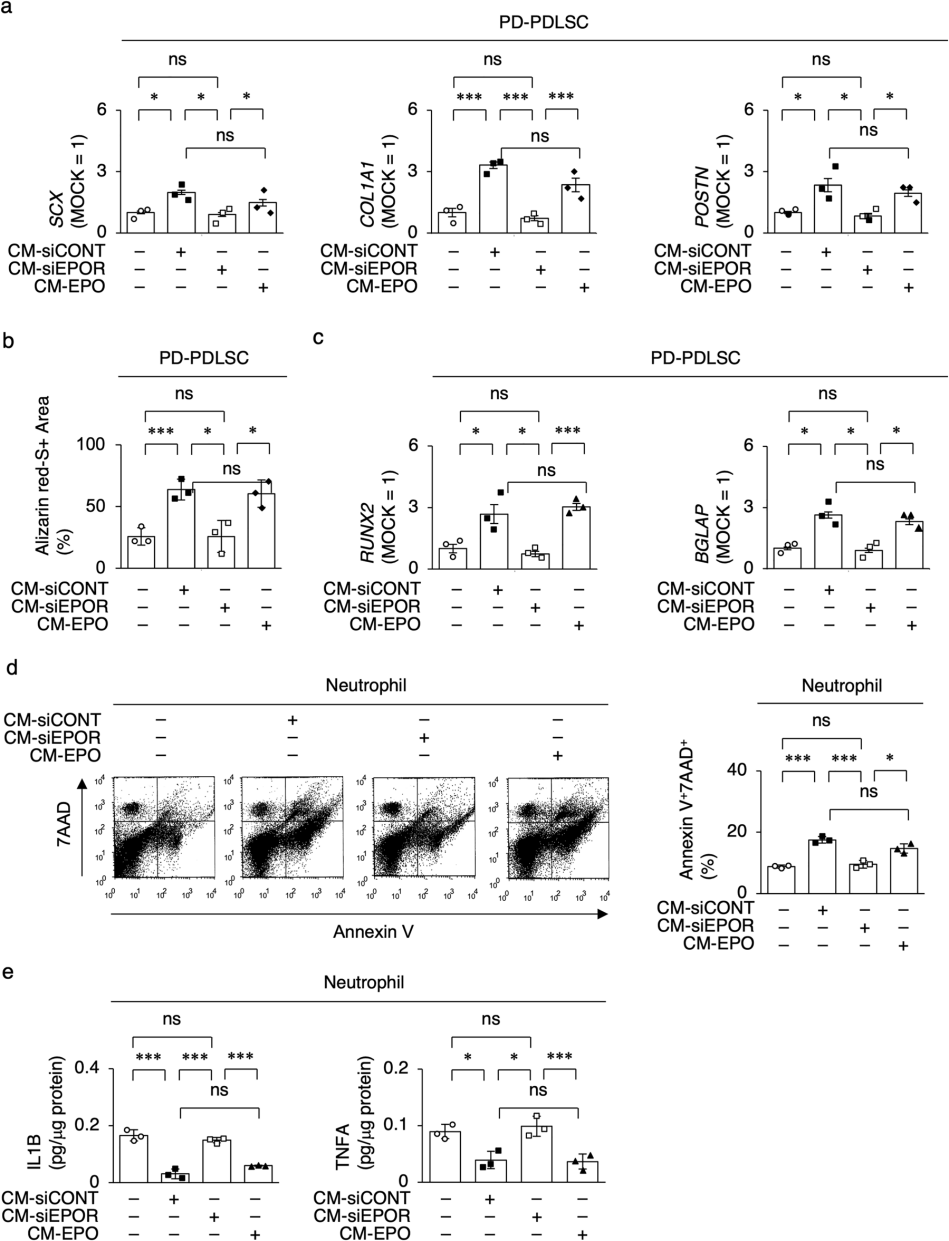
EPOR signal control paracrine effects of PDLSCs on ligamentogenic and cementogenic/osteogenic differentiation and neutrophil modulation. (**a**) Expression of *SCX*, *COL1A1*, and *POSTN* in ligamentogenic PD-PDLSCs by RT-qPCR. (**b**) Ratio (%) of Alizarin Res-S-positive area of cementogenic/osteogenic PD-PDLSCs. (**c**) Expression of *RUNX2* and *BGLAP* in cementogenic/osteogenic PD-PDLSCs by RT-qPCR. (**d**) Representative dot-blots of Annexin V^+^7AAD^+^ cells in LPS primed neutrophils by flow cytometry analysis. Annexin V^+^7AAD^+^ cells in LPS-primed neutrophils by flow cytometry analysis. (**e**) Levels of interleukin 1 beta (IL1B) and tumor necrosis factor-alpha (TNFA) in CM of LPS-primed neutrophils by ELISA. (**a–e**) CM-siCONT, CM of siCONT-PDLSCs: CM-siEPOR, CM of siEPOR-PDLSCs: CM-EPO, CM of EPO treated PD-PDLSCs. Data are presented as the mean ± SEM. *n* = 3/group. Significance was determined by two-way ANOVA with Tukey’s post hoc test; *P < 0.05 and ***P < 0.005. no significance (ns). (**a,c**) The results are shown as a ratio to the expression in the control group (MOCK = 1).

Given that the microenvironment-modulating functions, including the immunomodulatory and regenerative activity, are carried out by paracrine-released secretomes of MSCs^25^, the present results suggested that EPO/EPOR-regulated paracrine factors may contribute to the microenvironment-modulating functions of PDLSCs. Therefore, in this study, we focused on the roles of the conditioned medium (CM) of PDLSC cultures (CM-PDLSC) as an ideal tool for investigating the paracrine activity of PDLSCs. PD-PDLSCs were cultured under ligamentogenic and cementogenic/osteogenic conditions with CM-PDLSC stimulation. RT-qPCR and Alizarin Red-S staining revealed that the CM of siCONT-PDLSC cultures (CM-siCONT) increased the expression of *SCX*, *COL1A1*, and *POSTN* in PD-PDLSCs and in vitro mineralized nodule formation of PD-PDLSCs compared with non-CM treatment (Fig. 4a–c). CM of siEPOR-PDLSC cultures (CM-siEPOR) did not affect the in vitro ligamentogenic and cementogenic/osteogenic induction of PD-PDLSCs, whereas CM of EPO-PD-PDLSC cultures (CM-EPO) enhanced the in vitro ligamentogenic and cementogenic/osteogenic capacities of PD-PDLSCs compared to CM of MOCK-PDLSC cultures (CM-MOCK) (Fig. 4a–c).

Neutrophils are the most important cells that contribute to host defense and tissue damage in periodontal tissue^26^. Human neutrophils were primed with lipopolysaccharide (LPS) in the presence or absence of CM-PDLSC for 1 h. Flow cytometry showed that CM-siCONT induced Annexin V^+^7AAD^+^ cells in LPS-primed neutrophils compared to non-CM, whereas CM-siEPOR did not. CM-EPO induced Annexin V^+^7AAD^+^ cells (Fig. 4d). ELISA showed that CM-Cont and CM-EPO suppressed interleukin 1 beta (IL1B) and tumor necrosis factor-alpha (TNFA) levels in the CM of neutrophil cultures, whereas CM-siEPOR did not (Fig. 4e). These finding suggested that EPO/EPOR-regulated paracrine factors of PDLSCs participates in the micro-environmental function of PDLSCs.

### EPOR signal-regulated paracrine activity of PDLSCs regulates osteogenesis and osteoclastogenesis via ABSCs under oxidative stress

Periodontal inflammation is also accompanied by oxidative stress^26^. H_2_O_2_ suppressed *EPOR* and *TERT* expression and telomerase activity in Cont-PDLSCs (Fig. S3a–c). H_2_O_2_ damaged in vitro ligamentogenic and cementogenic properties and in vitro immunomodulatory function of Cont-PDLSCs (Fig. S3d–g). Whereas EPO treatment recovered the dysfunctions of H_2_O_2_ preconditioned Cont-PDLSCs (Fig. S3). Healthy donor-derived alveolar bone mesenchymal stem cells (Cont-ABSCs) exhibited colony formation, immunophenotype, and in vitro multipotency (Fig. S4a, b). H_2_O_2_ preconditioned Cont-ABSCs (H_2_O_2_-ABSCs) exhibited a poorer immunophenotype, reduced multipotency, and less immunosuppression compared to MOCK (PBS) preconditioned Cont-ABSCs (MOCK-ABSCs) (Fig. S4c–e). These findings suggested that H_2_O_2_ preconditioning, at least partially, mimics an oxidative stress environment in periodontitis. Thus, in this study, H_2_O_2_ preconditioning was used as a suitable model for investigating the function of periodontal disease-specific PDLSCs and ABSCs.

**Figure 5:**
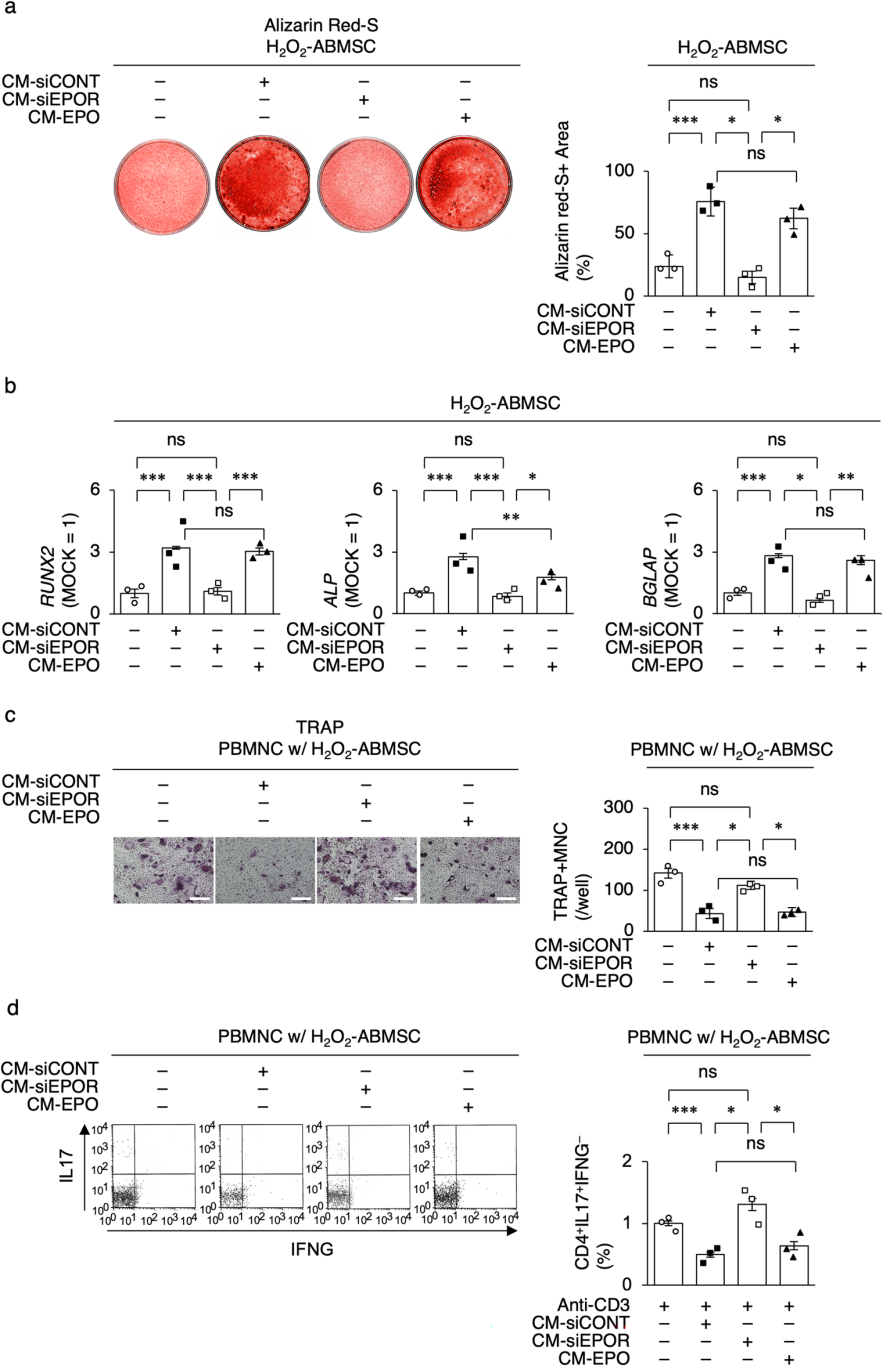
EPOR signal regulates anti-bone loss effects of PDLSCs on H_2_O_2_-damaged human alveolar bone stem cells. (**a**) Representative images of mineralized nodules of osteogenic H_2_O_2_-preconditioned human alveolar bone mesenchymal stem cells (H_2_O_2_-ABSCs) by Alizarin Res-S staining. Scale bars, 100 mm. Ratio (%) of Alizarin Red-S-positive area of osteogenic H_2_O_2_-ABSCs. (**b**) Expression of *RUNX2*, *alkaline phosphatase* (*ALP*), and *BGLAP* in osteogenic H_2_O_2_-ABSCs by RT-qPCR. The results are shown as a ratio to the expression in the control group (MOCK = 1). (**c**) Representative images of human PBMNCs co-cultured with H_2_O_2_-ABSCs (PBMNC w/H_2_O_2_-ABSC) under vitamin D_3_ stimulation in the presence of CM of PDLSC by tartrate-resistant acid phosphatase (TRAP) staining. White scale bars, 100 mm. Number of TRAP-positive multinuclear cells (TRAP + MNCs) per well. (**d**) Ratio of CD4^+^IL17^+^interferon gamma^–^ (CD4^+^IL17^+^IFNG^–^) cells in plate-bounded anti-CD3e antibody (Anti-CD3) activated human PBMNCs co-cultured with H_2_O_2_-ABSCs (PBMNC w/ H_2_O_2_-ABSC) under transforming growth factor and interleukin 6 (IL6) stimulation in the presence of CM of PDLSC by flow cytometry analysis. (**a–d**) CM-siCONT, CM of siCONT-PDLSCs; CM-siEPOR, CM of siEPOR-PDLSCs; CM-EPO, CM of EPO treated PD-PDLSCs. Data are presented as the mean ± SEM. *n* = 3/group. Significance was determined by two-way ANOVA with Tukey’s post hoc test; *P < 0.05, **P < 0.01, and ***P < 0.005. ns: no significance.

Oxidative stress suppresses the osteogenic function and increases the osteoclastogenic capacity of BMMSCs^27^. To investigate the paracrine activity of PDLSCs, the osteogenic capacity of H_2_O_2_-ABSCs was then analyzed under CM-PDLSC stimulation. CM-siCONT increased the in vitro mineralized tissue formation of H_2_O_2_-ABSCs, whereas CM-siEPOR did not, compared to MOCK (PBS) by Alizarin Red-S staining (Fig. 5a). CM-EPO enhanced the in vitro mineralized nodule formation in H_2_O_2_-ABSCs (Fig. 5a). The results of *RUNX2*, *ALP*, and *BGLAP* expression in H_2_O_2_-ABSCs under CM-PDLSC stimulation by RT-qPCR paralleled to that of in vitro mineralized tissue formation by Alizarin Red S staining (Fig. 5b). The effects of CM-PDLSC on osteoclastogenesis were also examined in a co-culture of human PBMNCs and H_2_O_2_-ABSCs under vitamin D_3_ stimulation. CM-siCONT reduced the formation of osteoclast-like tartrate-resistant acid phosphatase (TRAP) positive multinuclear cells (TRAP-positive MNCs), whereas CM-siEPOR did not, compared to the MOCK group (Fig. 5c). CM-EPO suppressed TRAP-positive MNC formation (Fig. 5c). the immunomodulatory effects of CM-PDLSC on IL17-releasing helper T (Th17) cell induction were examined by flow cytometry in a co-culture of anti-CD3e antibody-activated human PBMNCs and H_2_O_2_-ABSCs under transforming growth factor beta1 (TGFB1) and IL6 co-stimulation. CM-siCONT suppressed the induction of Th17 CD4^+^IL17^+^ interferon gamma^–^ (IFNG^–^) cells, whereas CM-siEPOR did not, compared to the MOCK group (Fig. 5d). CM-EPO inhibited the induction of CD4^+^IL17^+^IFNG^–^ cells (Fig. 5d). Thus, these results suggest that the EPOR signal-regulated paracrine activity of PDLSCs is involved in the bone recovery of the periodontal disease environment.

### EPOR signal contributes to alveolar bone regeneration in PDLSC-based therapy in periodontitis model mice

**Figure 6:**
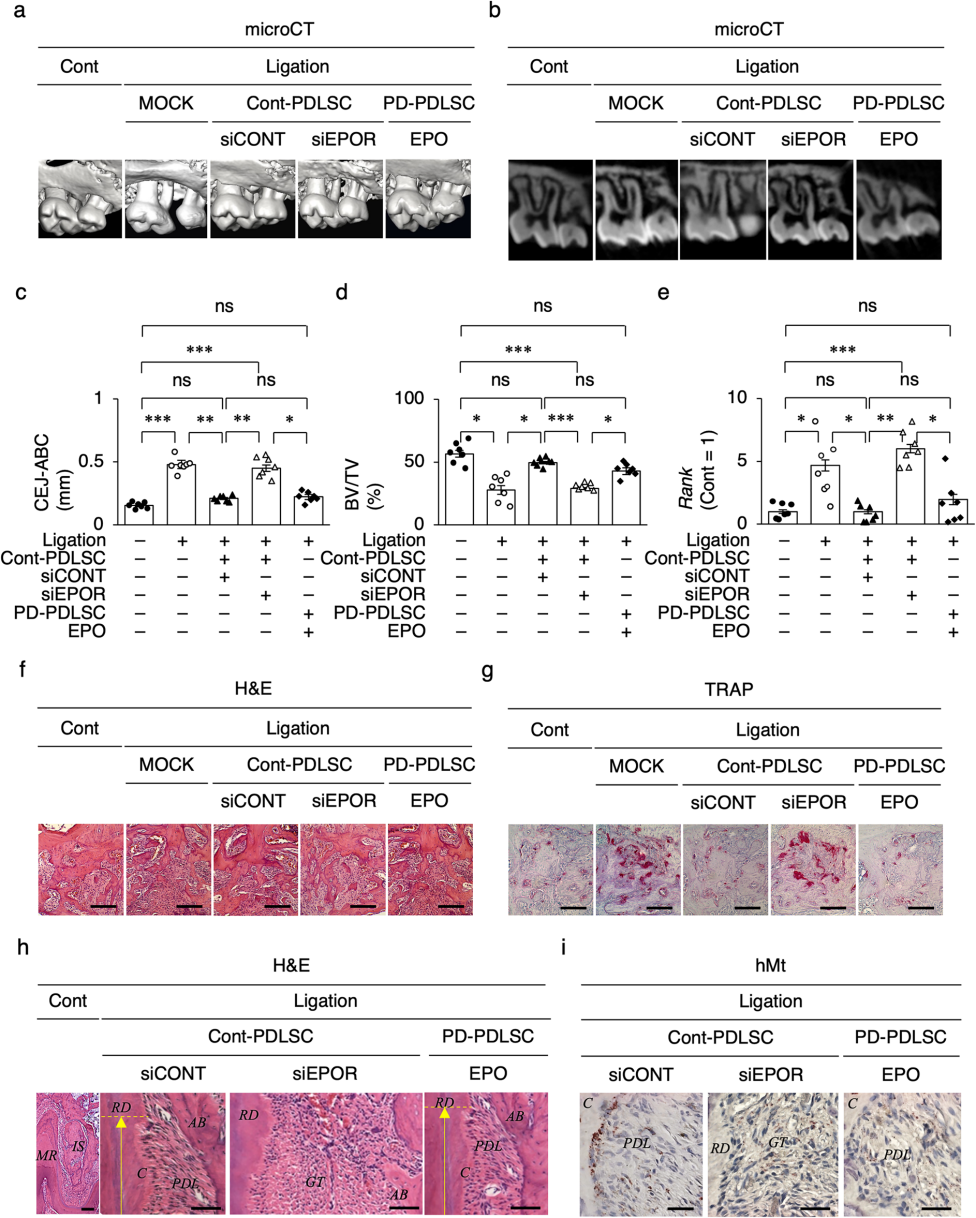
EPOR signal regulates alveolar bone regeneration in PDLSC-based therapy. (**a,b**) Representative three-dimensional (**a**) and two-dimensional (**b**) micro-CT images of alveolar bone. (**c**) Levels of the cementoenamel junction-alveolar bone crest (CEJ-ABC) in mesial buccal tooth roots of the maxillary second molar. (**d**) Bone volume per total volume (BV/TV) of alveolar bone around maxillary second molars. (**e**) Expression of *Rank* in gingival tissue around maxillary second molars by RT-qPCR. The results are shown as a ratio to the expression in the control group (Cont = 1). (**f,g**) Representative histological images of bone tissue of interradicular septa in the secondary molars by H&E (**f**) and TRAP (**g**) staining. (**h**) Representative histological images of periodontal tissue around mesial buccal tooth roots of maxillary second molar by H&E staining. *AB* alveolar bone, *C* cementum, *IS* interradicular septa, *MR* mesial buccal root, *PDL* periodontal ligament, *RD* root dentin. Yellow arrow area, resorbed root area. (**i**) Representative images of the distribution of human mitochondria (hMt) positive cells around the mesial tooth roots by immunohistochemical analysis. Toluidine blue counter-staining. (**a–i**) Cont, non-ligation group; ligation, ligation induced periodontitis group; MOCK, PBS administration group; Cont-PDLSC, Cont-PDLSC transplantation group; PD-PDLSC, PD-PDLSC transplantation group; siCONT, siCONT pretreatment; siEPOR, siEPOR pretreatment; EPO, EPO pretreatment. (**c–e**) Data are presented as the mean ± SEM. *n* = 7/group. Significance was determined by two-way ANOVA with Tukey’s post hoc test; *P < 0.05, **P < 0.01, and ***P < 0.005. *ns* no significance. (**f–i**) Scale bars, 100 mm.

To understand the importance of the EPOR signal in PDLSC-based therapy for periodontal disease, PDLSCs were locally transplanted around the maxillary second molars in ligature-induced periodontitis model mice 7 days after ligation and the periodontal bone condition was assessed 14 days after transplantation. Microcomputed tomography (micro-CT) analysis showed that the MOCK-infused non-PDLSC transplantation (non-Tx) induced substantial alveolar bone loss around the maxillary second molars and increased the levels of the cementoenamel junction-alveolar bone crest (CEJ-ABC) around the mesial buccal root of the second molar compared to the non-ligation control group (Fig. 6a–d). Local transplantation of siCONT-PDLSCs (siCONT-PDLSC-Tx) recovered the alveolar bone loss and CEJ-ABC levels around the second molar, whereas local transplantation of siEPOR-PDLSCs (siEPOR-PDLSC-Tx) failed (Fig. 6a–d). Local transplantation of EPO-PD-PDLSCs (EPO-PD-PDLSC-Tx) succeeded in the periodontal bone recovery around the second molar (Fig. 6a–d). RT-qPCR showed that local non-Tx enhanced the gingival *receptor activator of nuclear factor κB* (*Rank*) expression, which contributed to osteoclastic bone loss in periodontitis and oxidative stress^27,28^ Local siCONT-PDLSC-Tx suppressed gingival *Rank* expression around the maxillary second molar, whereas local siEPOR-PDLSC-Tx did not, compared to the non-Tx group (Fig. 6e). Local EPO-PD-PDLSC-Tx suppressed gingival *Rank* expression around the second molar (Fig. 6e). The results of the alveolar bone loss condition around the mesial buccal root of the second molar by histological analysis using H&E staining correlated with that by micro-CT analysis in all transplant groups (Fig. 6f). TRAP staining revealed that local siCONT-PDLSC-Tx reduced the expression of TRAP-positive cells in the periodontal bone lesions compared with non-Tx (Fig. 6g). Local siEPOR-PDLSC-Tx did not affect TRAP-positive expression, whereas local EPO-PD-PDLSC-Tx suppressed TRAP-positive cells in the periodontal alveolar bone (Fig. 6g). Further histological analysis revealed that the granular tissue was filled in bone loss lesions around tooth roots of the maxillary second molars in the non-TX group (Fig. 6h). Local siCONT-PDLSC-Tx induced de novo PDL-associated cementum on the resorbed tooth root surface and de novo alveolar bone, whereas local siEPOR-PDLSC-Tx did not (Fig. 6h). Local EPO-PD-PDLSC-Tx exhibited the periodontal regeneration as seen in local siCONT-PDLSC-Tx (Fig. 6h). Immunohistochemical analysis demonstrated that human mitochondria-positive cells were abundantly found on the surface of the de novo cementum and in the PDL of the siCONT-PDLSC-Tx and EPO-PD-PDLSC-Tx groups, whereas human mitochondria-positive cells were scattered in the granular tissues around tooth roots in the siEPO-PDLSC-Tx group (Fig. 6i).

### EPOR function regulates paracrine immune suppression in PDLSC-based therapy in periodontitis model mice

**Figure 7:**
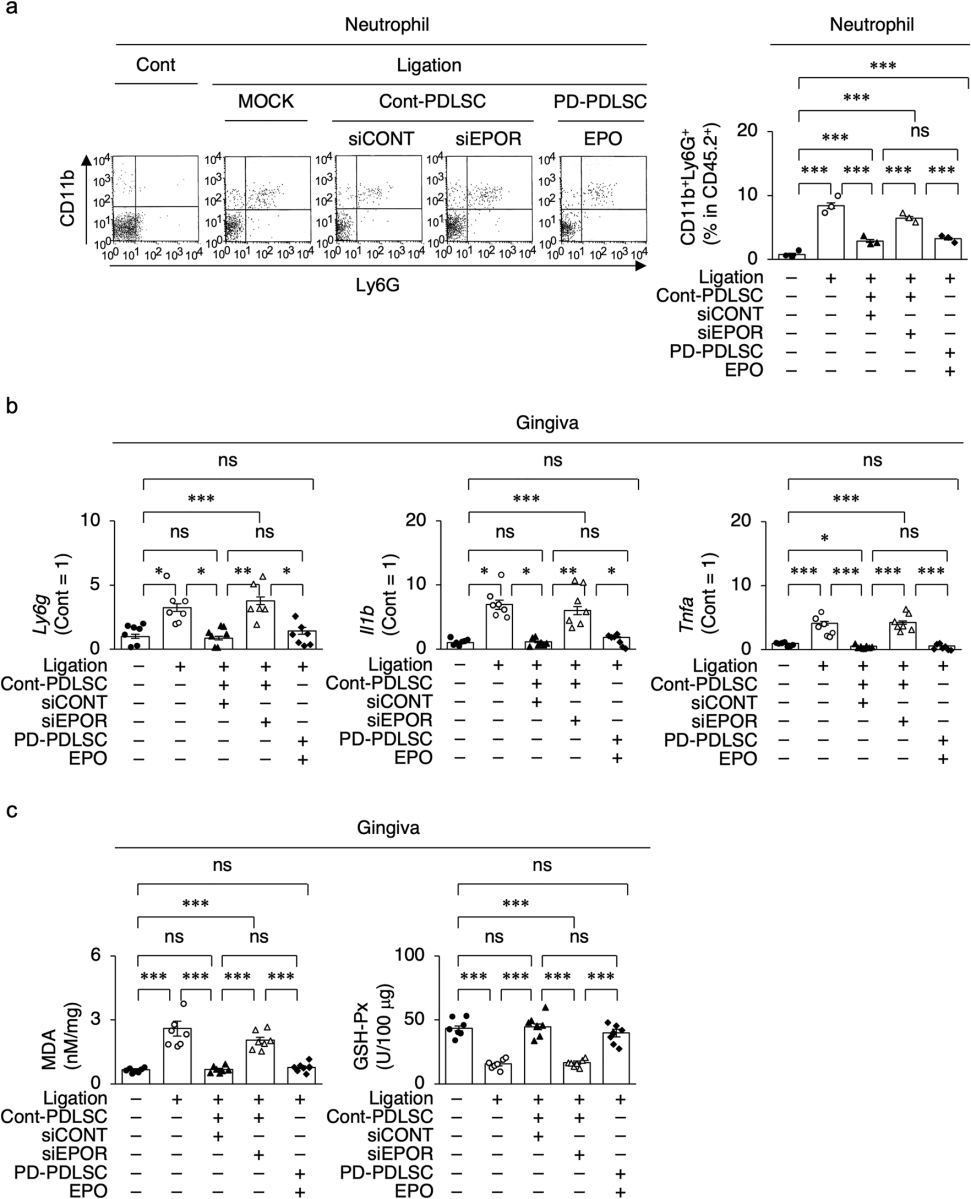
EPOR signal regulates periodontal immune modulation in PDLSC-based therapy. (**a**) Representative dot-blots of gingival CD45.2^+^CD11b^+^Ly6G^+^ cells by flow cytometry analysis. CD45.2^+^CD11b^+^Ly6G^+^ cells in gingival tissue around maxillary second molars by flow cytometry analysis. (**b**) Expression of *Ly6g*, *Il1b*, *Tnfa* in gingiva around maxillary second molars by RT-qPCR. The results are shown as a ratio to the expression in the control group (Cont = 1). (**c**) Malondialdehyde (MDA) levels and glutathione peroxidase (GSH-Px) activity in gingiva around maxillary second molars by colorimetric analysis. (**a–c**) Cont, non-ligation group; ligation, ligation induced periodontitis group; MOCK, PBS administration group; Cont-PDLSC, Cont-PDLSC transplantation group; PD-PDLSC, PD-PDLSC transplantation group; siCONT, siCONT pretreatment; siEPOR, siEPOR pretreatment; EPO, EPO pretreatment. Data are presented as the mean ± SEM. *n* = 7/group. Significance was determined by two-way ANOVA with Tukey’s post hoc test; *P < 0.05, **P < 0.01, and ***P < 0.005. *ns* no significance.

Finally, we investigated the immunological and reactive oxygen species (ROS) conditions in the gingival lesions around the maxillary second molars in the ligature-induced periodontitis mouse model. Flow cytometry demonstrated that neutrophil-like CD45.2^+^CD11b^+^ lymphocyte antigen 6 complex locus^+^ (Ly6G^+^) cells were increased in the inflamed gingival lesions of the non-Tx group compared with the control group (Fig. 7a). Local siCONT-PDLSC-Tx reduced the gingival CD45.2^+^CD11b^+^Ly6G^+^ cells around the maxillary second molars, whereas local siEPOR-PDLSC-Tx did not (Fig. 7a). Local EPO-PD-PDLSC-Tx suppressed the gingival CD45.2^+^CD11b^+^Ly6G^+^ cells as seen in local siCONT-PDLSC-Tx (Fig. 7a). RT-qPCR showed that non-Tx enhanced the gingival levels of neutrophil-related genes, including *Ly6g*, *Il1b*, and *Tnfa* (Fig. 7b), as well as T cell-related genes, including *CD3e*, *retinoic acid receptor-related orphan receptor gamma* (*Rorgc*), *Tgfb1*, *Il6*, *Il17*, and *Il22* (Fig. S5), around the maxillary second molars compared to the control group. Local siCONT-PDLSC-Tx reduced the gingival expression of neutrophil- and T cell-related genes, whereas local siEPOR-PDLSC-Tx did not (Fig. 7b, Fig. S5). RT-qPCR showed that local EPO-PD-PDLSC-Tx improved the abnormal gingival expression of neutrophil- and T-cell-related genes, whereas siEPOR-PDLSC-Tx did not (Fig. 7b, Fig. S5). Colorimetric assays showed that non-Tx enhanced the gingival ROS production, as indicated by the increased level of malondialdehyde (MDA) and suppressed activity of glutathione peroxidase (GSH-Px), compared to the control group. (Fig. 7c). Local siCONT-PDLSC-Tx and local EPO-PD-PDLSC-Tx improved the abnormal gingival levels of MDA and GSH-Px, whereas local siEPOR-PDLSC-Tx did not (Fig. 7c). Thus, these results suggest that the EPOR signal-regulated microenvironment-modulating function is considerably involved in the recovery of the periodontal environment in PDLSC-based therapy.

## Discussion

The microenvironment-modulating functions of tissue-specific stem cells contribute crucially to tissue homeostasis, repair, and regeneration^29^. The significance of EPOR/STAT5 signaling is understood in the microenvironment-modulating functions of hematopoietic stem cells and erythroid progenitors in the body^30^ and the importance of STAT5 signaling in erythroid differentiation has been well evaluated^31,32^. The deletion of STAT5 in erythroid progenitors results in anemia^33^. However, the role of EPOR/STAT5 signaling has been unclear in the microenvironment-modulating functions of non-hematopoietic stem/progenitor cells such as MSCs. Here, we demonstrated that the silencing of functional EPOR in healthy PDLSCs damaged microenvironment-modulating functions, including stemness, multipotency, and immunomodulatory function, mimicking the characteristics of periodontal disease-specific and ROS-damaged pathological PDLSCs. Recently, a negative feedback loop of EPO-stimulated STAT5 phosphorylation was shown to suppress the cell surface expression of EPOR via ubiquitination-mediated endocytosis under physiological red blood differentiation^34,35^, suggesting that the downregulation of EPOR provides a defensive mechanism against erythroid hyperproliferation caused by the underlying excessive receptor signaling in primary familial and congenital polycythemia. However, it is hypothesized that another positive feedback loop mechanism rejuvenates the tissue stem cells in a pathological environment, as demonstrated by the present results, in which EPO stimulation induced enhanced expression of the EPOR gene and protein and phosphorylated STAT5 as well as the recovery of microenvironment-modulating dysfunction in pathological PDLSCs.

The microenvironment-modulating functions of MSCs are largely mediated by the secretion of trophic factors^29^. The paracrine action of healthy PDLSCs governs periodontal bone loss^36,37^, whereas pathological PDLSCs display fewer anti-bone loss paracrine effects than healthy PDLSCs^38^. The present manipulations of EPOR function through gene silencing and ligand-induced activation demonstrate the contribution of EPOR signaling in the paracrine action of pathological PDLSCs to the periodontal tissue reconstruction of PDL, cementum, and alveolar bone. Given that BMMSCs reconstruct hematopoietic tissues in a multifaceted manner via paracrine action^18,39,40^, the present results suggest that EPOR signaling contributes significantly to the regulation of the orchestral paracrine action of PDLSCs as a microenvironment modulator for tissue reconstruction in cell-based periodontal therapy. However, the direct EPO preconditioning effects on PDLSCs cannot be avoided compared to the indirect paracrine actions in PDLSC-based periodontal therapy. Further studies are necessary to determine the critical trophic factors in PDLSCs that are controlled by EPO/EPOR signals.

Many studies indicate that orofacial pathological conditions cause epigenetic damage to orofacial tissue-specific stem cells^41–44^. In periodontal lesions, infiltrating hyper-reactive neutrophils release abundant ROS, leading to periodontal disease associated with connective tissue destruction, alveolar bone resorption, and tooth loss^26^, indicating that periodontal ROS acts as a factor responsible for periodontal disease. In this study, H_2_O_2_ exposure suppresses *EPOR* and *TERT* expression, telomerase activity, ligamentogenic and cementogenic/osteogenic potency, and immunosuppressive function in healthy PDLSCs, which mimicked the pathological PDLSCs. Indeed, H_2_O_2_ exposure causes cellular dysfunctions of healthy ABSCs, including impaired osteoblast differentiation, increased osteoclast induction, and suppressed immunosuppression. These results indicate that periodontal ROS epigenetically facilitates the storage of disease-specific memories in PDLSCs via EPOR signaling. Thus, these findings suggest that an EPOR signal-controlled antineutrophil factor may be a critical target for periodontal tissue reconstruction in PDLSC-based therapies.

Self-renewal and multipotency are the most important properties of tissue-specific MSCs^45,46^. The stemness of MSCs must be sustained to maintain homeostasis by recruiting tissue-specific cells when tissue is damaged in the human body^47^. Stemness is maintained under telomerase activity^48^. Telomerase reactivation in adult stem cells recovers tissue damage^49^, whereas telomerase impairment causes epigenetic changes in stem cells^50^. Telomerase activity in cells is controlled by the essential catalytic subunit TERT^51^. TERT-mediated telomerase activity is considered significant for regulating the microenvironment-modulating function of tissue-specific MSCs^40,48,52^. EPO-exposed EPOR/STAT5 signaling transcriptionally regulates TERT gene expression in leukemia cell lines^53^. Given the present findings from EPO-exposed pathological PDLSCs, it is suggested that EPO-mediated EPOR signaling targets impaired stemness in pathological PDLSCs to rejuvenate the paracrine microenvironment-modulating action in autologous disease-specific PDLSC-based periodontal therapy.

Current preclinical studies have demonstrated that the autologous transplantation of PDL progenitors and PDLSCs is a feasible option for alveolar bone recovery in periodontitis^54–56^. Given the limited availability of healthy PDLSCs from patients with periodontitis, the clinical benefits of autologous PDLSC-based periodontal therapy seem to be limited^1^. Disease-specific PDLSCs are alternative candidates for autologous cell-based periodontal therapy. However, recent studies have demonstrated that disease-specific PDLSCs fail to achieve direct tissue regeneration and indirect microenvironmental modulating functions^57–59^, suggesting that autologous disease-specific PDLSCs have insufficient clinical outcomes. Current studies have revealed that in vitro and in vivo pharmacological approaches are acceptable options for rejuvenating the damaged functions of disease-specific tissue stem cells^41,43,60^. Recombinant EPO is clinically used to treat secondary anemia in patients with chronic kidney disease and cancer^61^. EPO/EPOR signaling regulates epigenetic changes in the survival, proliferation, and differentiation of neural progenitor cells^62^. We demonstrated that exogenous EPO treatment is a feasible epigenetic approach for rejuvenating *EPOR* reductant and stem cell deficiency of disease-specific PDLSCs, leading to enhanced microenvironmental modulation of disease-specific PDLSCs via EPO/EPOR/STAT5 signaling, as correlated in BMMSCs^18^. Thus, considering our preclinical results, epigenetic activation of EPOR signaling may provide a new strategy to compensate for autologous PDLSC-based strategies for periodontal disease.

## Methods

### Ethics approval and consent to participate

All human samples were obtained from three independent healthy donors (19–23 years old) and three independent donors with periodontal disease (43–65 years old) with written informed consent at the Department of Pediatric Dentistry of Kyushu University Hospital. The procedures for using human samples were conducted following the Declaration of Helsinki. The procedures for handling human samples were approved by the Kyushu University Institutional Review Board for Human Genome/Gene Research on 15th March 2021 (Protocol Number: 738-03). All the animal experiments were approved by the Institutional Animal Care and Use Committee of Kyushu University on 10th January 2023 and 3rd February 2023 (Protocol Number: A23-028-0 and A23-074-0). All the experimental methods in this study were performed in accordance with the relevant guidelines and regulations and reported in accordance with ARRIVE guidelines.

### Animals

NOD/SCID mice (female, 7 weeks old) and Balb/c mice (female, 10 weeks old) were purchased from Charles River Laboratories, Japan (Yokohama, Japan). The animals were housed freely in sterile water and standard chow under controlled environmental conditions with a 12-h light/12-h dark cycle. The animals were sacrificed by cervical dislocation following systemic anesthesia with intraperitoneal injection of medetomidine hydrochloride (0.3 mg/kg), midazolam (4 mg/kg), and butorphanol tartrate (5 mg/kg).

### Antibodies and TaqMan probes

Antibodies and TaqMan probes used in this study were listed in Table S1–S3.

### Isolation and culture of PDLSCs and ABSCs

Following tooth extraction at Kyushu University Hospital, the healthy teeth and alveolar bone tips of systemically healthy donors (n = 3; 19–23 years old) were obtained due to the impaction of third molars with periodontally healthy clinically. The teeth with severe chronic periodontitis of systemically healthy donors (n = 3; 43–65 years old) were obtained due to the irreversible severe periodontitis associated with gingival inflammation, deep pocket depth, and high tooth mobility. The clinical tooth information of donors was summarized in Table S4. All the tooth samples were extracted at Kyushu University Hospital. Healthy and periodontal disease (PD)-derived PDL tissues were obtained from the apical 1/3 region of the tooth root surface of healthy and PD-derived samples. All the PDL samples were subjected to isolate PDLSCs using the colony-forming units-fibroblast (CFU-F) method as previously described^8,63^. Healthy ABSCs were also isolated from the alveolar bone tip samples using the CFU-F method as previously described^8,63^. All the samples were digested with an enzyme cocktail solution for 60 min at 37 °C. The enzyme cocktail consisted of 0.3% collagenase type I (Worthington Biochemicals, Lakewood, NJ, USA) and 0.4% dispase II (Sanko Junyaku, Tokyo, Japan) in sterilized Ca^2+^- and Mg^2+^-free phosphate-buffered saline (PBS). The digested tissue fragments were passed through a 70 μm cell strainer, and single suspended cells were seeded in T-75 flasks. The cultures were washed with PBS 24 h after seeding and subsequently cultured for 16 days in a complete cell growth medium (CGM). The CGM consisted of 15% fetal bovine serum (FBS; Equitech-Bio, Kerrville, TX), 100 μM l-ascorbic acid 2-phosphate (FUJIFILM Wako Pure Chemicals, Osaka, Japan), 2 mM l-glutamine (Nacalai Tesque, Kyoto, Japan), and a premixed antibiotic solution containing 100 U/mL penicillin, 100 μg/mL streptomycin, and 25 μg/mL amphotericin B (Nacalai Tesque) in Eagle’s minimum essential medium alpha modification (Thermo Fisher Scientific, Waltham, MA, USA). Attached colony-forming cells were passaged at 1.5 × 10^5^ per dish on 100 mm culture dishes. The expanded passaged 3 cells were characterized as MSCs according to a previous report^64^. The passaged 3 Cont-PDLSCs, PD-PDLSCs, and ABSCs were used for further experiments. Some PDLSCs and ABSCs were pretreated with H_2_O_2_ (125 nM; Nacalai Tesque) or PBS alone in CGM for 48 h and used.

### Functional knockdown and pharmacological activation of EPOR in PDLSCs

Cont-PDLSCs were treated for three days with siEPOR and siCONT (20 nM; Santa Cruz Biotechnology, Santa Cruz, CA, USA) using Lipofectamine RNAiMax (Thermo Fisher Scientific) according to the manufacturer’s instructions and used as siEPOR-PDLSCs and siCONT-PDLSCs. PD-PDLSCs were treated with recombinant human EPO (0.1 U/mL; R&D Systems, Minneapolis, MN, USA) or MOCK (PBS) for 2 days and used as EPO-PD-PDLSCs and MOCK-PD-PDLSCs. The effects of functional knockdown and pharmacological activation of EPOR were confirmed by immunoblotting and flow cytometry.

### Gene and protein expression of EPOR in PDLSCs

PDLSCs, including Cont-PDLSCs, PD-PDLSCs, MOCK-PD-PDLSCs, EPO-PD-PDLSCs, siCONT-PDLSCs, and siEPOR-PDLSCs, were cultured. RT-qPCR analyzed the expression of *EPOR* in PDLSCs. Immunoblotting assayed the expression of *EPOR* in PDLSCs. Phosphorylation levels of STAT5 in PDLSCs were temporally analyzed by immunoblot analysis 12 h after serum depletion. Immunofluorescent analysis analyzed the localization of EPOR in PDLSCs. Briefly. The cells were seeded in 2-well chamber slides (Thermo Fisher Scientific) and fixed with 4% paraformaldehyde (MilliporeSigma) in PBS (pH 7.4) for 15 min at room temperature. They were treated with 10% normal goat serum and incubated with EPOR-specific antibody or control IgG overnight at 4 °C. After washed with PBS, the samples were treated with Alexa Fluor 594-conjugated donkey anti-mouse antibody (Thermo Fisher Scientific), followed by staining with 4',6-diamidino-2-phenylindole (Thermo Fisher Scientific). Finally, the cells were observed under an Axio Imager M2 fluorescent up-light microscopy (Carl Zeiss Microscopy). Flow cytometry assayed the surface expression of EPOR in PDLSCs. PDLSCs (1.0 × 10^5^ per 100 µL) were resuspended in a flow cytometry buffer (FCB) at 4 °C. The FCB consisted of 2% heat-inactivated FBS (MilliporeSigma) in Hanks’ balanced salt solution (Nacalai Tesque). The cells were incubated with R-phycoerythrin-labeled specific antibodies to EPOR (1 mg/mL; R&D Systems, Minneapolis, MN, USA) or subclass-matched control antibodies (1 mg/mL; BioLegend) for 45 min at 4 °C and washed with FCB. All samples were measured using a FACSVerse flow cytometer (BD Bioscience) and analyzed using FACSuite software (BD Bioscience). Jurkat cells (Thermo Fisher Scientific) were used as a positive control for EPOR expression in RT-qPCR, immunoblotting, and immunofluorescent analysis.

### Preparation and treatment of CM-PDLSC

PDLSCs (2 × 10^5^ per 100 mm dish), including siCONT-PDLSCs, siEPOR-PDLSCs, and EPO-PD-PDLSCs, were cultured with CGM for 24 h. After washed 3 times with PBS, the cells were incubated with serum-free Dulbecco’s modified eagle medium (Nacalai Tesque) for 48 h. The culture supernatants were collected and kept at –20 °C until used. The samples were quickly thawed at 37 °C and centrifuged at 1500×*g* for 30 min. The supernatants were used as CM-PDLSC, including CM-siCONT, CM-siEPO, and CM-EPO. The CM-PDLSC and its MOCK (Dulbecco’s modified eagle medium alone) were enriched approximately tenfold using a Centriprep centrifugal filter (MilliporeSigma) according to the manufacturer’s instructions. The enriched CM-PDLSC and its MOCK were mixed with culture medium at a ratio of 1:9 and used for further experiments.

### CFU-F assay of PDLSCs and ABSCs

PDLSCs were seeded at 0.1, 1.0, and 10 × 10^6^ cells in 100 mm culture dishes and cultured in CGM. Fourteen days after seeding, the cultures were treated with 2% paraformaldehyde (MilliporeSigma, Burlington, MA, USA) and 0.1% toluidine blue (MilliporeSigma) in PBS. The stained samples were washed with distilled water three times and air-dried. Attached colonies were determined as cell clusters containing > 50 cells and the colony number was counted under a Primovert inverted microscope (Carl Zeiss Microscopy, Jena, Germany).

### Immunophenotype assay of PDLSCs and ABSCs

The expression of MSC cell surface markers, including CD146, CD105, CD73, CD34, CD45, and CD14, was examined on Cont-PDLSCs, PD-PDLSCs, and ABSCs by flow cytometry. PDLSCs and ABSCs (1.0 × 10^5^ per 100 µL) were resuspended in a flow cytometry buffer (FCB) at 4 °C. The FCB consisted of 2% heat-inactivated FBS (MilliporeSigma) in Hanks’ balanced salt solution (Nacalai Tesque). The cells were incubated with R-phycoerythrin-labeled specific antibodies to cell surface markers (1 mg/mL; BioLegend, San Diego, CA, USA) or subclass-matched control antibodies (1 mg/mL; BioLegend) for 45 min at 4 °C and washed with FCB. All samples were measured using a FACSVerse flow cytometer (BD Bioscience) and analyzed using FACSuite software (BD Bioscience).

### Population doubling assay of PDLSCs

Cont-PDLSCs and PD-PDLSCs were initially seeded at 2 × 10^5^ cells on a T-75 flask and cultured under CGM. The medium was changed twice a week. The cells were harvested and measured the harvest cells. They were passed at 2 × 10^5^ cells per flask and cultured for 1 week as described above. The passaging of PDLSCs was stopped until the cells lost the proliferative capacity. Population doubling level (PDL) at each passage was calculated by using a formula: PDL = 3.322 × (log [harvested cell number] – log [initial seeded cell number]) and total PDL was obtained.

### TERT gene expression and telomerase activity assays in PDLSCs

PDLSCs, including Cont-PDLSCs, PD-PDLSCs, MOCK-PD-PDLSCs, EPO-PD-PDLSCs, siCONT-PDLSCs, and siEPOR-PDLSCs, were cultured sed for analyzing of *TERT* expression by RT-qPCR and telomerase activity by TRAP-qPCR and ELISA using a TeloTAGGG telomerase PCR ELISA kit (MilliporeSigma) according to the manufacturer’s instructions. The results of telomerase activity were measured using a MULTISKAN GO spectrophotometer (Thermo Fisher Scientific). HEK293T cells (Thermo Fisher Scientific) and heat-inactivated samples were used as positive and negative controls for telomerase activity, respectively.

### BrdU incorporation assay of PDLSCs

For BrdU incorporation assay, PDLSCs (1 × 10^3^), including Cont-PDLSCs, PD-PDLSCs, MOCK-PD-PDLSCs, EPO-PD-PDLSCs, siCONT-PDLSCs, and siEPOR-PDLSCs, were cultured on a 2-well chamber slide (Thermo Fisher Scientific) in CGM for 2 days and examined by using a BrdU staining kit (Thermo Fisher Scientific) according to the manufacturer’s instructions. The nuclei were lightly stained with hematoxylin. The cell images were captured under an Axio Imager M2 upright microscope (Carl Zeiss Microscopy) and the ratio of BrdU-positive nuclei was quantified using ImageJ software (National Institutes of Health).

### In vitro T cell suppression assay of PDLSCs and ABSCs

Human PBMNCs (iQ Bioscience, Alameda, CA, USA) were activated at 1 × 10^6^ cells/well with plate bounded anti-CD3e antibody (1 mg/mL; APA1/1 clone; BioLegend) and soluble anti-CD28 antibody (1 mg/mL; CD28.2 clone; BioLegend) in a complete leukocyte medium (CLM) for 3 days. The CLM consisted of 5% heat-inactivated FBS (MilliporeSigma) and 100 U/mL penicillin, 100 μg/mL streptomycin, and 25 μg/mL amphotericin B (Nacalai Tesque) in RPMI-1640 medium (Nacalai Tesque). For T cell survival assay, some activated PBMNCs (1 × 10^5^ cells/well) were co-cultured with PDLSCs (1 × 10^5^ cells/well), including Cont-PDLSCs, PD-PDLSCs, MOCK-PD-PDLSCs, EPO-PD-PDLSCs, siCONT-PDLSCs, siEPOR-PDLSCs. Some Cont-PDLSCs and ABSCs were preconditioned with and without H_2_O_2_ and used for the co-culture in the presence and absence of human recombinant EPO in CLM for 3 days on 24-well plates. The viability of the floating cells was analyzed by colorimetric assay using a Cell Counting Reagent SF (Nacalai Tesque) according to the manufacturer’s instructions. The results of cell viability were measured using a MULTISKAN GO spectrophotometer (Thermo Fisher Scientific).

For Th17 cell suppression assay, the other activated PBMNCs (1 × 10^5^ cells/well) were co-cultured with H_2_O_2_-preconditioned ABSCs (1 × 10^5^ cells/well) under the stimulation with 2 mg/mL human TGFB1 (PeproTech) and 50 mg/mL IL6 (PeproTech) for 3.5 days in the presence or absence of CM-PDLSC, including CM-siCONT, CM-siEPO, and CM-EPO, in CLM. The floating cells (1.0 × 10^5^ per 100 µL) were resuspended in FCB at 4 °C and incubated with R-phycoerythrin-labeled specific antibodies to human IL17 and human IFNG (1 mg/mL each; Biolegend) or subclass-matched control antibodies for 45 min at 4 °C and washed with FCB. All samples were measured for CD4^+^IL17^+^IFNG^–^ cells as Th17 cells using a FACSVerse flow cytometer (BD Bioscience) and analyzed using FACSuite software (BD Bioscience).

### In vitro adipogenic, chondrogenic, cementogenic/osteogenic, and ligamentogenic induction assays of PDLSCs and ABSCs

PDLSCs, including Cont-PDLSCs, PD-PDLSCs, MOCK-PD-PDLSCs, EPO-PD-PDLSCs, siCONT-PDLSCs, and ABSCs preconditioned with and without H_2_O_2_ (5 × 10^3^/dish) were cultured on 35 mm culture dishes at a confluent condition in the CGM and induced under specific induction conditions for adipocytes, chondrocytes, cementoblasts/osteoblasts, and ligament cells. The induction mediums were changed twice a week.

For the adipogenic induction assay, the cells were incubated for 6 weeks in an adipocyte-induction medium consisting of CGM supplemented with 500 mM isobutyl-methylxanthine (MilliporeSigma), 60 mM indomethacin (MilliporeSigma), 0.5 mM hydrocortisone (MilliporeSigma), and 10 mM insulin (MilliporeSigma). The cultures were stained with 0.3% Oil Red O (MilliporeSigma) and analyzed lipid droplet formation under a Primovert inverted microscope (Carl Zeiss Microscopy). Expression of adipocyte-specific genes, including *peroxisome proliferator-activated receptor gamma* and *lipoprotein lipase*, were also analyzed by RT-qPCR.

For the chondrogenic induction assay, the cells were incubated for 4 weeks in a chondrocyte-induction medium consisting of CGM supplemented with 10 ng/mL TGFB1 (Peprotech, Rocky Hill, NJ, USA), 1% ITS premix (BD Bioscience, Franklin Lake, NJ, USA), 100 nM dexamethasone (MilliporeSigma), and 2 mM sodium pyruvate (Nacalai Tesque). The cultures were stained with 0.2% Alcian blue (MilliporeSigma) and analyzed cartilage matrix formation under a Primovert inverted microscope (Carl Zeiss Microscopy). Expression of chondrocyte-specific genes, including *SRY-box transcription factor 9* and *aggrecan*, were also analyzed by RT-qPCR.

For the cementogenic/osteogenic induction assay, the cells were incubated in a cementoblast/osteoblast-induction medium consisting of CGM supplemented with 1.8 mM potassium dihydrogen phosphate (MilliporeSigma), 10 nM dexamethasone (MilliporeSigma), 2 mM l-glutamine (Nacalai Tesque). The cultures were stained with 1% Alizarin Red S (MilliporeSigma) 4 weeks after cementogenic/osteogenic induction and analyzed calcium deposition formation. The mineralized area (Alizarin Red-S-positive area per total area) was measured using Image J software (National Institutes of Health, Bethesda, MD, USA). Expression of cementoblast/osteoblast-specific genes, including *RUNX2*, *alkaline phosphatase* (*ALP*), and *BGLAP*, were also analyzed by RT-qPCR. Gene expression and secretion of SEMA3A in PDLSCs and CM-PDLSC were analyzed by RT-qPCR and ELISA. Total protein concentration in CM-PDLSC of leukocyte cultures was determined using a Pierce Protein Assay kit (Thermo Fisher Scientific) according to the manufacturer’s instructions. The concentration of SEMA3A in CM-PDLSC was measured by ELISA using Human SEMA3A ELISA Kit (LifeSpan Biosciences, Seattle, WA, USA) according to the manufacturer’s instructions. The concentrations of total protein and cytokines are measured using a MULTISKAN GO spectrophotometer (Thermo Fisher Scientific). SEMA3A levels were normalized against the total protein concentrations.

For the ligamentogenic induction assay, the cells were incubated for 4 weeks in a ligament-cell-induction medium consisting of the CGM with 10 nM TGFB3 (Peprotech). The cultures were stained with Picro-Sirius Red (Abcam, Cambridge, UK) and analyzed collagen matrix formation. Expression of ligament-cell-specific genes, including *COL1A1*, *POSTN*, and *SCX*, were also analyzed by RT-qPCR.

### In vivo tissue regenerative assay of PDLSCs

PDLSCs, including Cont-PDLSCs, PD-PDLSCs, MOCK-PD-PDLSCs, EPO-PD-PDLSCs, siCONT-PDLSCs, and ABSCs preconditioned with and without H_2_O_2_ were cultured in CGM until sub-confluent condition. The present in vivo tissue regenerative assay of PDLSCs was performed according to a previous study^8^. PDLSCs and ABSCs (4.0 × 10^6^ each) were mixed with 40 mg hydroxyapatite/tricalcium phosphate powder as a carrier (Zimmer Inc., Warsaw, IN, USA) for 90 min at 37 °C. Immunocompromised NOD/SCID mice (10-week-old) were systemically anesthetized by intraperitoneal injection of medetomidine hydrochloride (0.3 mg/kg), midazolam (4 mg/kg), and butorphanol tartrate (5 mg/kg). The mixtures of the cells and carrier were subcutaneously implanted into the back skin of NOD/SCID mice. The mice were subsequently kept on a heating pad at 37 °C until they recovered and subcutaneously injected with buprenorphine (0.02 mg/kg) for pain relief. Eight weeks after implantation, the implant tissues were harvested. The tissue samples were fixed with 4% paraformaldehyde (MilliporeSigma) in PBS (pH 7.4) overnight at 4 °C and decalcified with 5% ethylenediaminetetraacetic acid (EDTA) solution (pH 8.2). For histological analysis, some samples were dehydrated and embedded in paraffin and the paraffin sections were stained with H&E (MilliporeSigma). For immunohistochemical analysis, the other samples were cut using a CM1950 cryostat (Leica Biosystems, Wetzlar, Germany). The cryosections were treated with 10% normal mouse serum (Vector Laboratories, Newark, CA, USA) for 30 min and incubated with human mitochondria antibody-specific antibody or control IgG. The sections were treated with a VECTASTAIN ABC-HRP kit, Peroxidase (Mouse IgG) (Vector Laboratories) according to the manufacturer’s instructions and lightly counterstained with hematoxylin (MilliporeSigma). All sections were observed using an Axio Imager M2 up-light microscopy (Carl Zeiss Microscopy).

### Human neutrophil suppression assay of CM-PDLSCs

Neutrophils were isolated from human PBMNCs (iQ Bioscience) using an EasySep™ Direct Human Neutrophil Isolation Kit (STEMCELL Technologies, Vancouver, Canada) according to the manufacturer's instructions as previously described^65^. Neutrophils (1 × 10^6^ cells/well) were primed with or without LPS (100 ng/mL; MilliporeSigma) for 1 h in the presence or absence of CM-PDLSC in CLM. The floating cells were stained using an APC Annexin-V Apoptosis Detection Kit with 7-AAD (BioLegend, San Diego, CA, USA) and analyzed apoptotic cells by flow cytometry using a FACSVerse flow cytometer (BD Bioscience) and analyzed using FACSuite software (BD Bioscience). The CMs of the neutrophil cultures were assayed for human IL1B and TNFA by ELISA using LEGEND MAX Human IL-1beta ELISA Kit (BioLegend) and LEGEND MAX Human TNF-α ELISA Kit (BioLegend). The results are measured using a MULTISKAN GO spectrophotometer (Thermo Fisher Scientific).

### In vitro human osteoclast formation assay in PBMNC-ABSC coculture system

Human osteoclasts were induced in the coculture system of PBMNCs and ABSCs as previously described^66^. Briefly, ABSCs (5 × 10^3^ cells/well) were incubated overnight in 24 well plates with CGM for 48 h. Human PBMNCs (iQ Bioscience, Alameda, CA, USA; 3 × 10^5^/well) were co-cultured with H_2_O_2_-preconditioned ABSCs in an osteoclastogenic induction medium. The osteoclast induction medium consisted of 10% FBS (MilliporeSigma), 10 nM 1α,25(OH)_2_D_3_ (FUJIFILM Wako Pure Chemical Industries), 10 nM dexamethasone (MilliporeSigma), 25 ng/mL macrophage colony-stimulating factor (PeproTech, Cranvery, NJ, USA), and 100 U/mL penicillin, 100 μg/mL streptomycin, and 25 μg/mL amphotericin B (Nacalai Tesque) in Eagle’s minimum essential medium alpha modification (Thermo Fisher Scientific). The whole culture medium was changed every three days. Osteoclast formation was determined by tartrate-resistant acid phosphatase (TRAP) staining using an Acid Phosphatase, Leukocyte (TRAP) kit (MilliporeSigma) according to the manufacturer’s instructions. TRAP-positive multinuclear cells (MNCs) (> 3 nuclei) were counted using a Primovert inverted microscope (Carl Zeiss Microscopy).

### Local transplantation of PDLSCs in ligature-induced periodontal disease model mice

Recently, a ligature-induced periodontal disease model was established according to a previous protocol^67^. Recent studies indicate that BALB/cJ mice exhibit more susceptibility to *Porphyromonas gingivalis* compared to C57BL/6 J mice^68,69^. We employed BALB/cJ mice for establishing a ligature-induced periodontal disease model in this study. Briefly, a silk suture (#5–0; Roboz Surgical Instrument Co., MD, USA) was ligated around the right maxillary second molars of Balb/cJ mice (10-week-old). As transplant donors, PDLSCs, including siCONT-PDLSCs, siEPOR-PDLSCs, and EPO-PD-PDLSCs, were cultured in CGM. Seven days after ligation, each PDLSC suspension (1 × 10^5^ in 20 μL PBS; *n* = 22–24 per each donor) or PBS (*n* = 23) was submucosally infused under the palatal gingiva of maxillary second molars using a Hamilton syringe equipped with a 33-gauge needle (Hamilton Company, Reno, NV, USA). The gingivae around the left maxillary second molar were used as negative controls. Throughout the surgery, all animals were anesthetized by intraperitoneal injection of medetomidine hydrochloride (0.3 mg/kg), midazolam (4 mg/kg), and butorphanol tartrate (5 mg/kg). The post-operated mice were subsequently kept on a heating pad at 37 °C until they recovered. All mice were inspected daily and mice with lost ligatures (*n* = 1–3 per each donor/group) were excluded from the study. Maxillae of the mice were harvested en bloc 14 days after cell infusion and quickly removed the mucosal tissues. The samples per group were randomly selected to use for further study.

### Mouse alveolar bone assays

Mouse maxillary bone samples (*n* = 14 per group) were fixed with 4% paraformaldehyde (MilliporeSigma) in PBS (pH 7.4) overnight at 4 °C. Some bone samples (*n* = 7 per group) were used for micro-CT analysis using a micro-CT scanner 1076 micro-computed tomography system (Skyscan, Kontich, Belgium) and CTAn software (Skyscan). Alveolar bone loss was determined as the level of the cementoenamel junction-alveolar bone crest at the mesial buccal root region of the second molars according to a previous study^70^. The bone volume versus the total volume of alveolar bone around the maxillary second molars was analyzed. For histological analysis by hematoxylin and eosin, TRAP, and immunohistochemical staining, the other bone samples (*n* = 14 per group) were decalcified with 5% EDTA solution (pH 8.2). Some bone samples (*n* = 7 per group) were embedded in paraffin. Some paraffin sections were stained with hematoxylin and eosin solution (MilliporeSigma). For TRAP staining, the other sections were immersed in 50% acetone and 50% ethanol solution for 10 min at room temperature and treated using a TRAP staining solution. The TRAP staining solution was mixed with 9.6 mg of naphthol AS-BI phosphate (MilliporeSigma) in 0.6 mL of *N, N*-dimethylformamide (MilliporeSigma), and 84 mg of fast red-violet LB diazonium salt (MilliporeSigma), 58.2 mg of tartaric acid (MilliporeSigma), and 240 mL of 10% MgCl_2_ (MilliporeSigma) in 60 mL of 0.2 M sodium acetate buffer (pH 5.0). The sections were lightly counterstained with 0.1% toluidine blue solution (MilliporeSigma). For immunohistochemical staining, the other bone samples (*n* = 7 per group) were cut using a CM1950 cryostat (Leica Biosystems, Wetzlar, Germany). The cryosections were treated with 10% normal mouse serum (Vector Laboratories) for 30 min and incubated with human mitochondria antibody-specific antibody or control IgG. The sections were treated with a VECTASTAIN ABC-HRP kit, Peroxidase (Mouse IgG) (Vector Laboratories) according to the manufacturer’s instructions. All sections were observed using an Axio Imager M2 up-light microscopy (Carl Zeiss Microscopy).

### Mouse gingival assays

Mouse gingival tissues surrounding the maxillary molars (*n* = 7 per group) were digested with 1 mL tissue digestion buffer consisting of 2 mg/mL collagenase type II (Worthington Biochemicals, Lakewood, NJ, USA) and 1 mg/mL DNase Type I (Roche, Basel, Switzerland) in PBS for 20 min at 37 °C, followed by treatment with 10 mM EDTA for 10 min at 37 °C. Single-cell suspensions were obtained using a 40 μm cell-strainer. To detect mouse leukocytes, the gingival cells were labeled with Alexa 488-labeled anti-CD45.2, allophycocyanin-labeled anti-CD11b, and R-phycoerythrin-labeled lymphocyte antigen 6 complex locus antibodies. All samples were analyzed for CD45.2^+^CD11b^+^Ly6G^+^ cells using a FACSVerse flow cytometer (BD Bioscience) and analyzed using FACSuite software (BD Bioscience).

Total RNA and protein were extracted from the mouse gingival tissues of maxillary second molars (*n* = 7 per group for each extraction). Gene expression of *Ly6g*, *Il1b*, *Tnfa*, and *Rank* was analyzed by RT-qPCR. MDA level and GSH-Px activity were analyzed by colorimetric analysis using MDA assay kit (Dojindo, Kumamoto, Japan) and Glutathione Peroxidase (GSH-Px) Activity Kit (Thermo Fisher Scientific). The results are measured using a MULTISKAN GO spectrophotometer (Thermo Fisher Scientific).

### RT-qPCR

Samples were lysed in TRIzol reagent (Thermo Fisher Scientific), digested with RQ1 RNase-Free DNase I (Promega, Madison, WI, USA), and cleaned using an RNeasy Mini Kit (Qiagen, Valencia, CA, USA). cDNA was prepared by reverse transcription using a Revertra Ace qPCR kit (TOYOBO, Osaka, Japan) according to the manufacturer’s instructions. RT-qPCR was subsequently performed using TaqMan Gene Expression Master Mix (Thermo Fisher Scientific) and target TaqMan probes (Thermo Fisher Scientific) with a Light Cycler 96 system real-time PCR machine (Roche, Penzberg, Germany). Human and mouse 18S ribosomal RNAs were used for normalization.

### Immunoblot analysis

Samples were lysed in M-PER mammalian protein extraction reagent (Thermo Fisher Scientific) supplemented with a proteinase inhibitor cocktail (Nacalai Tesque) and phosphatase inhibitor PhoSTOP (Roche). They were separated using TGX FastCast acrylamide gels (Bio-Rad Laboratories, Hercules, CA, USA) and transferred using a Trans-Blot Turbo Transfer System (Bio-Rad Laboratories). The membranes were blocked with 5% skim milk in Tris-buffered saline (150 mM NaCl and 20 mM Tris–HCl, pH 7.2) for 1 h at room temperature and then incubated with primary antibodies overnight at 4 °C. The membranes were then treated with horseradish peroxidase-conjugated donkey anti-rabbit secondary antibody (1:1000; Santa Cruz Biotechnology, Santa Cruz, CA, USA) for 1 h at room temperature. Each membrane was treated with WB Stripping Solution Strong (Nacalai Tesque) and stained with anti-actin beta antibody (MilliporeSigma), followed by incubation with horseradish peroxidase-conjugated donkey anti-mouse IgG secondary antibody (1:1000; Santa Cruz Biotechnology). The bound antibodies were visualized using SuperSignal West Pico (Thermo Fisher Scientific) on an Image Quant LAS 4010 imager (GE Healthcare Life Sciences, Pittsburgh, PA, USA). ACTB was used as an internal control.

### Statistical analysis

All samples were selected randomly. The in vitro results obtained using each donor PDLSCs per group were used as the average of at least three repeated tests for statistical analysis. The in vivo results were randomly obtained from seven mice per group for statistical analysis. All data are shown as the mean ± standard error of the mean (SEM). Comparisons between the two groups were analyzed using an independent two-tailed Student’s *t*-test. Multi-group comparisons were analyzed using a one-way repeated measures analysis of variance followed by Tukey’s post hoc test. P-values less than 0.05 were considered statistically significant. All statistical analyses were analyzed using the PRISM 9 software (GraphPad, Software, La Jolla, CA).

### Supplementary Information

Supplementary Figures.

Supplementary Tables.

## Data Availability

Data and materials supporting the findings of this study are available in the article and its Supplementary Tables and Figures.
