# Supplementary material for: Erythropoietin receptor signal is crucial for periodontal ligament stem cell-based tissue reconstruction in periodontal disease
Source: Sci Rep. 2024 Mar 20;14:6719. doi: 10.1038/s41598-024-57361-y (PMC10954634; doi:10.1038/s41598-024-57361-y)
Supplement: Supplementary file 1 — Supplementary Figures. [file 41598_2024_57361_MOESM1_ESM.pdf]

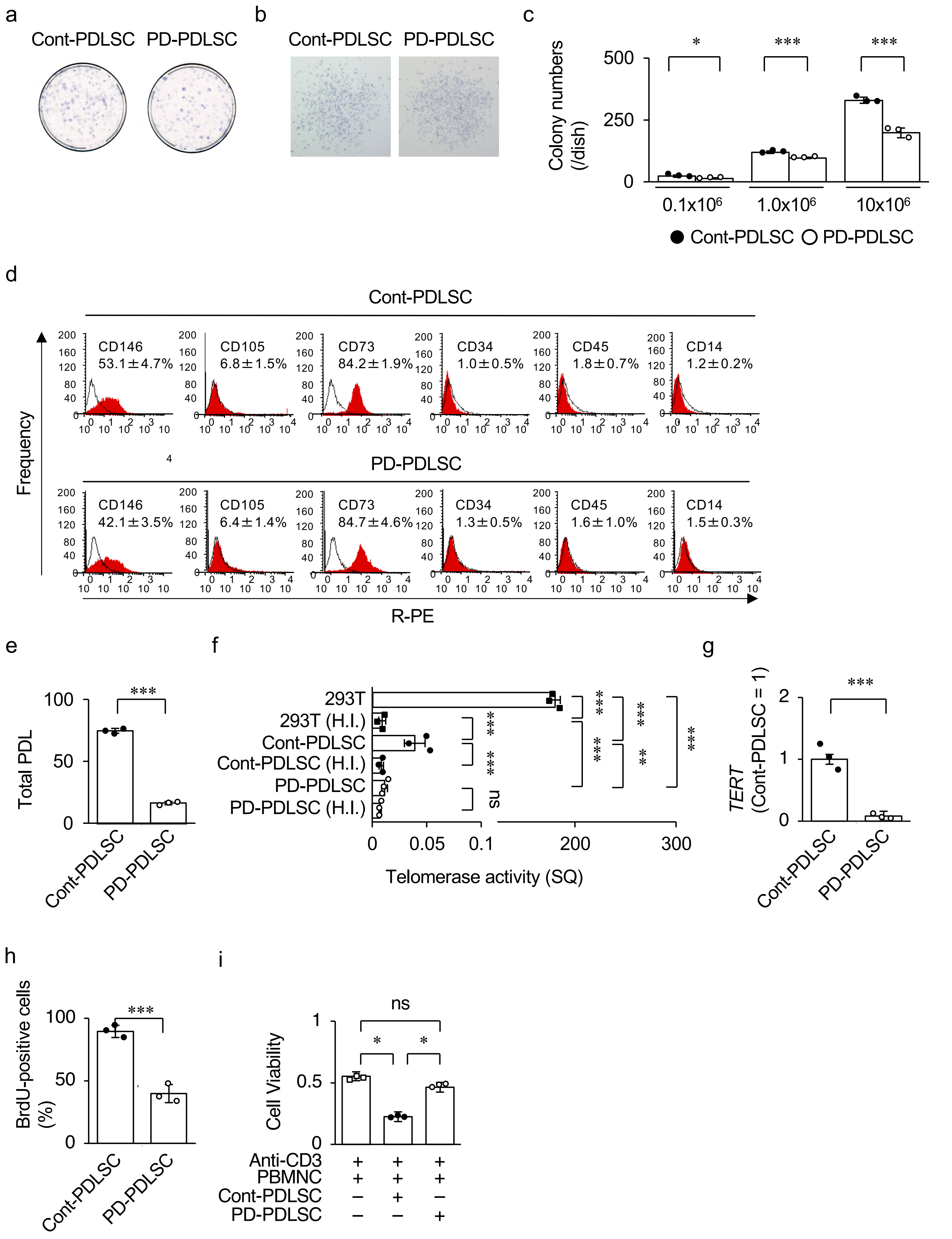

Fig. S1. Characterization of periodontitis-derived periodontal stem cells. This figure legend was mentioned on the following page.

Fig. S1. Characterization of periodontitis-derived periodontal stem cells. (**a, b**) Representative whole dish (**a**) and single colony (**b**) images of attached colony forming capacity of periodontitis-derived periodontal stem cells (PD-PDLSCs) compared to healthy-patient-derived PDLSCs (Cont-PDLSCs) by toluidine blue staining. (**c**) Number of attached colonies of PD-PDLSCs seeded at 0.1, 1.0, and 10 $\times 10^6$ cells. (**d**) Representative histograms of cell surface markers on PD-PDLSCs by flow cytometry (FCM). Positive rates (%) are presented as mean ± SEM. White area: histograms stained with control antibody; red area: histograms stained with antibodies against cell surface markers. R-PE, R-Phycoerythrin. (**e**) Population doubling level (total PDL) of PD-PDLSCs. (**f**) Telomerase activity of PD-PDLSCs by telomerase repeat amplification protocol and polymerase chain reaction (TRAP-PCR). 293T, HEK293T cells; H.I., heat-inactivated samples. SQ, threshold cycles. (**g**) Expression of *telomerase reverse transcriptase* (*TERT*) in PDLSCs by RT-qPCR. The results are shown as a ratio to the expression in Cont-PDLSCs (Cont-PDLSC = 1). (**h**) Bromodeoxyuridine (BrdU) incorporation capacity of PD-PDLSCs. (**i**) Cell viability of plate-bounded anti-CD3 epsilon (CD3e) antibody (Anti-CD3) activated human peripheral blood mononuclear cells (PBMNCs) co-cultured with Cont-PDLSCs and PD-PDLSCs. **c-i;** Data are presented as mean ± SEM. n = 3/group. **c, e-i**; Significance was determined by independent two-tailed Student's t test (**c, e, g, h**) and two-way ANOVA with Tukey's post hoc test (**f, i**); * $P < 0.05$, **$P < 0.01$, and *** $P < 0.005$. ns, no significance.

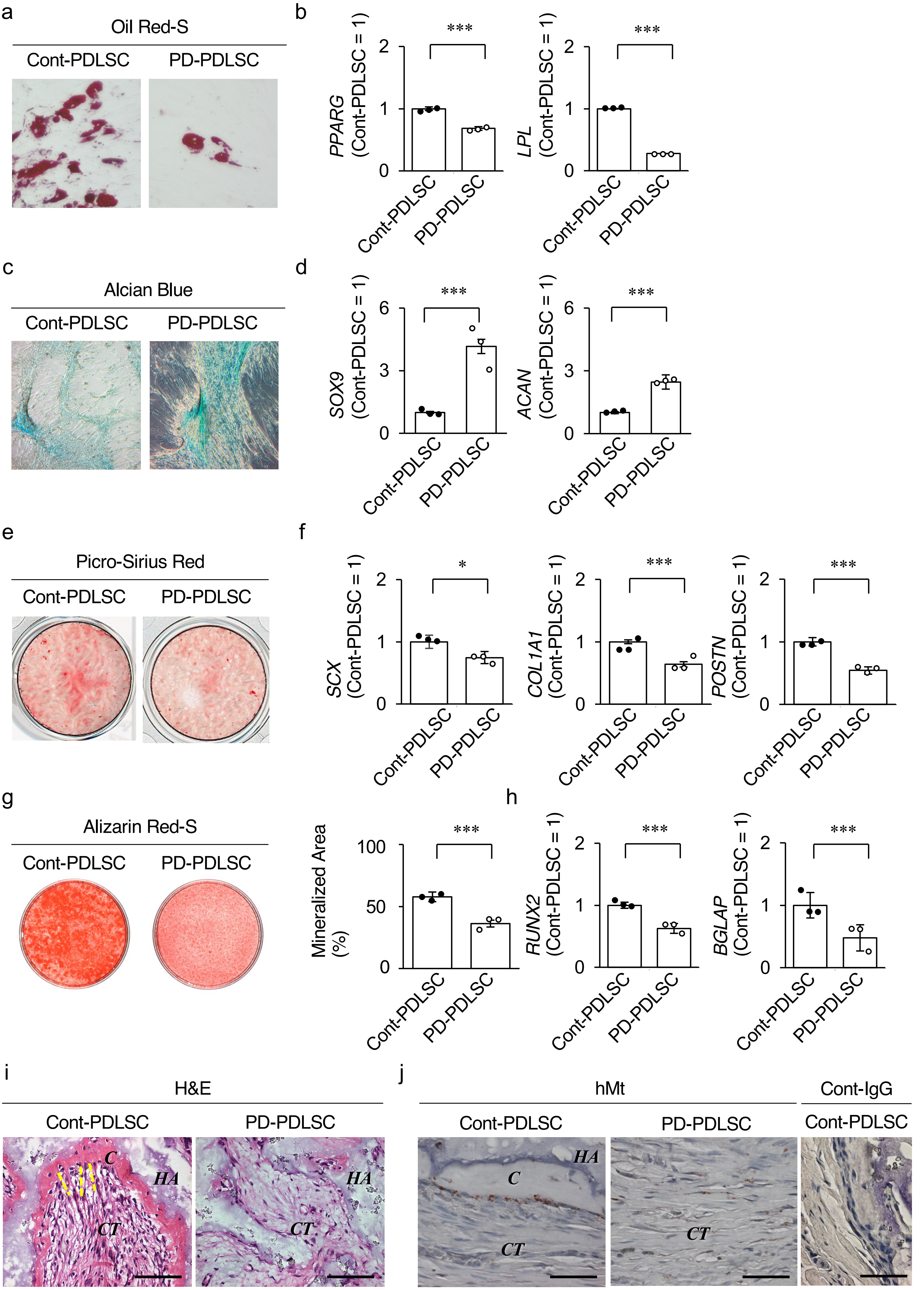

Fig. S2. ***In vitro*** **multipotency into adipocytes, chondrocytes, ligament cells, and cementoblasts/osteoblasts and** ***in vivo*** **periodontal tissue regeneration of PD-PDLSCs.** This figure legend was mentioned on the following page.

Fig. S2. *In vitro* multipotency into adipocytes, chondrocytes, ligament cells, and cementoblasts/osteoblasts and *in vivo* periodontal tissue regeneration of PD-PDLSCs. (**a**) Representative images of adipogenic PDLSCs by Oil Red O staining. (**b**) Expression of *lipoprotein lipase* (*LPL*) and *peroxisome proliferator-activated receptor gamma* (*PPARG*) of adipogenic PDLSCs by reverse transcription-quantitative polymerase chain reaction (RT-qPCR). (**c**) Representative images of chondrogenic PDLSCs by Alcian blue staining. (**d**) Expression of *SRY-box transcription factor 9* (*SOX9*) and *aggrecan* (*ACAN*) of chondrogenic PDLSCs by RT-qPCR. (**e**) Representative images of ligamentogenic PDLSCs by Picro-Sirius Red staining. (**f**) Expression of *collagen type I alpha 1 chain* (*COL1A1*), *periostin* (*POSTN*), and *scleraxis BHLH transcription factor* (*SCX*) in ligamnetogenic PDLSCs by RT-qPCR. (**g**) Representative images of cementogenic/osteogenic PDLSCs by Alizarin Red-S staining. Ratio (%) of Alizarin Red-S-positive area of mineralized nodules of cementogenic/osteogenic PDLSCs. (**h**) Expression of *runt-related family transcription factor 2* (*RUNX2*) and *bone gamma-carboxyglutaminate protein* (*BGLAP*) in cementogenic/osteogenic PDLSCs by RT-qPCR. (**i, j**) Representative histological images of subcutaneous implant tissues of PDLSCs by hematoxylin and eosin (H&E) staining (**i**) and immunohistochemical staining with anti-human mitochondria (hMt) antibody or control IgG (Cont-IgG) and hematoxylin counter staining (**j**). *C*, cementum-like mineralized tissue; *CT*, fibrous connective tissue; *HA,* hydroxyapatite and tricalcium phosphate. Yellow dot line, Sharpey's fiber-like structure (**i**). **b, d, f, h**: Data are presented as mean ± SEM. $n = 3$/group. Significance was determined by independent two-tailed Student's t test; *** $P < 0.005$. The results are shown as a ratio to the expression in Cont-PDLSCs (Cont-PDLSC = 1). **i, j**: Scale bar, 100 μm (**i**), 50 μm (**j**).

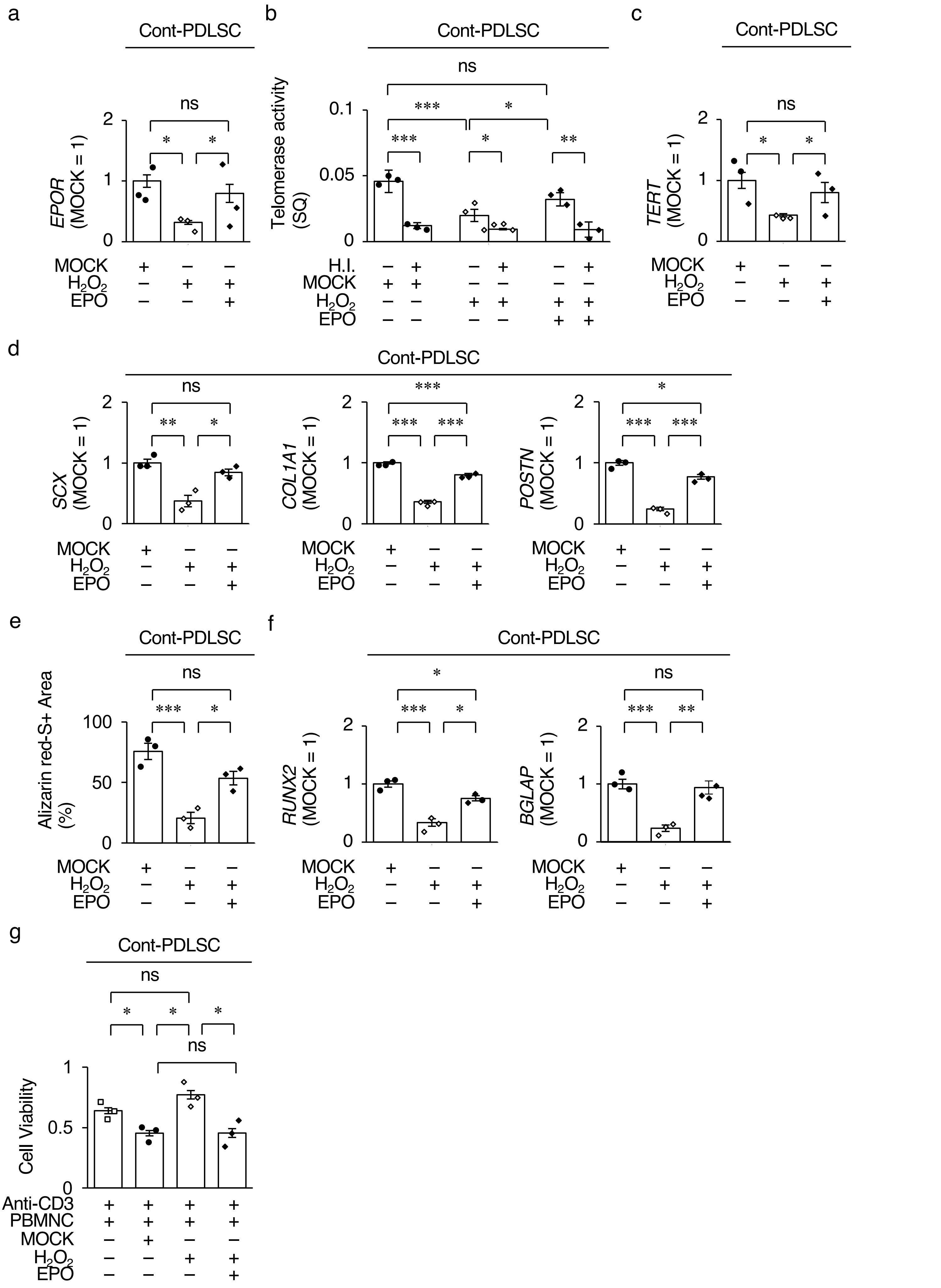

Fig. S3. Erythropoietin recovers EPOR expression, telomerase activity, *in vitro* ligamentogenic and cementogenic/osteogenic potency, and *in vitro* immunosuppressive function of human PDLSCs under oxidative stress condition. This figure legend was mentioned on the following page.

Fig. S3. Erythropoietin recovers EPOR expression, telomerase activity, *in vitro* ligamentogenic and cementogenic/osteogenic potency, and *in vitro* immunosuppressive function of human PDLSCs under oxidative stress condition. (**a**) Expression of *EPOR* in PDLSCs by RT-qPCR. (**b**) Telomerase activity of PDLSCs by TRAP-PCR. H.I., heat inactivated samples. SQ, threshold cycles. (**c**) Expression of *TERT* in PDLSCs by RT-qPCR. (**d**) Expression of *SCX*, *COL1A1*, and *POSTN* of ligamentogenic PDLSCs by RT-qPCR. (**e**) Alizarin Red-S-positive area of cementogenic/osteogenic PDLSCs. (**f**) Expression of *RUNX2* and *BGLAP* of cementogenic/osteogenic PDLSCs by RT-qPCR. (**g**) Cell viability of plate-bounded anti-CD3e antibody (Anti-CD3) activated PBMNCs co-cultured with Cont-PDLSCs. **a–g**: EPO, human recombinant erythropoietin treatment; H_2O_2, H_2O_2 treatment; MOCK, PBS treatment. Data are presented as mean ± SEM. $n = 3$/group. Significance was determined by two-way ANOVA with Tukey's post hoc test; * $P < 0.05$, ** $P < 0.01$, and *** $P < 0.005$. ns, no significance. **a, c, d, f**: The results are shown as a ratio to the expression in MOCK-treated Cont-PDLSCs (MOCK = 1).

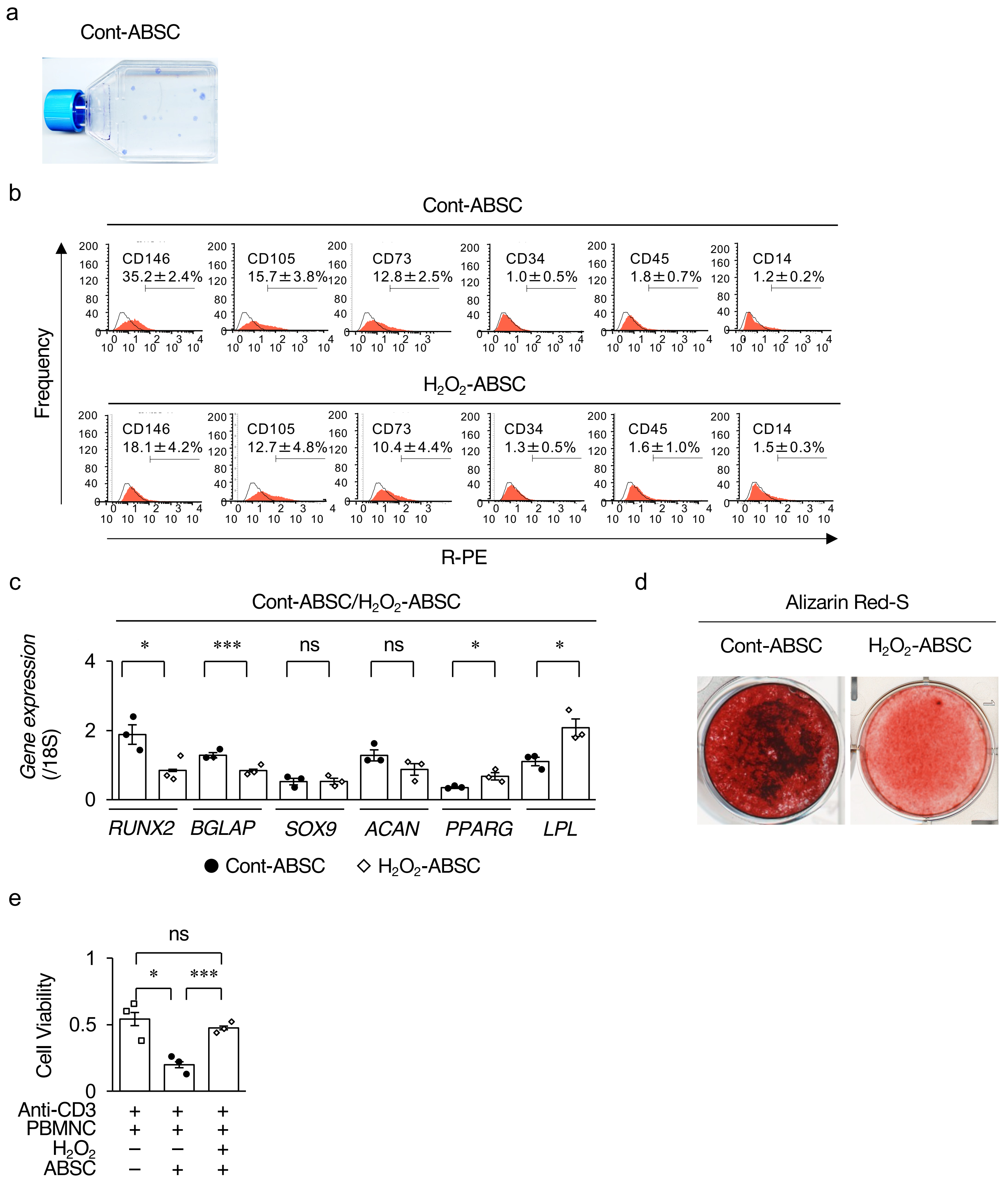

Fig. S4. Characterization and effects of oxidative stress on human alveolar bone mesenchymal stem cells. This figure legend was mentioned on the following page.

Fig. S4. Characterization and effects of oxidative stress on human alveolar bone mesenchymal stem cells. (**a**) Representative images of attached colony forming capacity of human alveolar bone stem cells (ABSCs). (**b**) Representative histograms of cell surface markers on ABSCs by FCM analysis. Positive rates (%) are presented as mean ± SEM. White area: histograms stained with control antibody; red area: histograms stained with antibodies against cell surface markers. R-PE, R-Phycoerythrin. (**c**) Expression of *RUNX2*, *BGLAP*, *SOX9*, *ACAN*, *PPARG*, and LPL of osteogenic, chondrogenic, and adipogenic PDLSCs by RT-qPCR. The results are shown as a ratio to the expression of 18S rRNA (/18S). (**d**) Representative images of mineralized nodules of osteogenic PDLSCs by Alizarin Red-S staining. (**e**) Cell viability of plate-bounded anti-CD3e antibody (Anti-CD3) activated human PBMNCs co-cultured with Cont-ABSCs and H_2O_2-ABSCs. **a–e**; Cont-ABSC, healthy donor derived ABSCs; H_2O_2-ABSC, H_2O_2 treated ABSCs. **c, e**; Data are presented as mean ± SEM. n = 3/group. Significance was determined by independent two-tailed Student's t test; $* P < 0.05$, $*** P < 0.005$. ns, no significance.

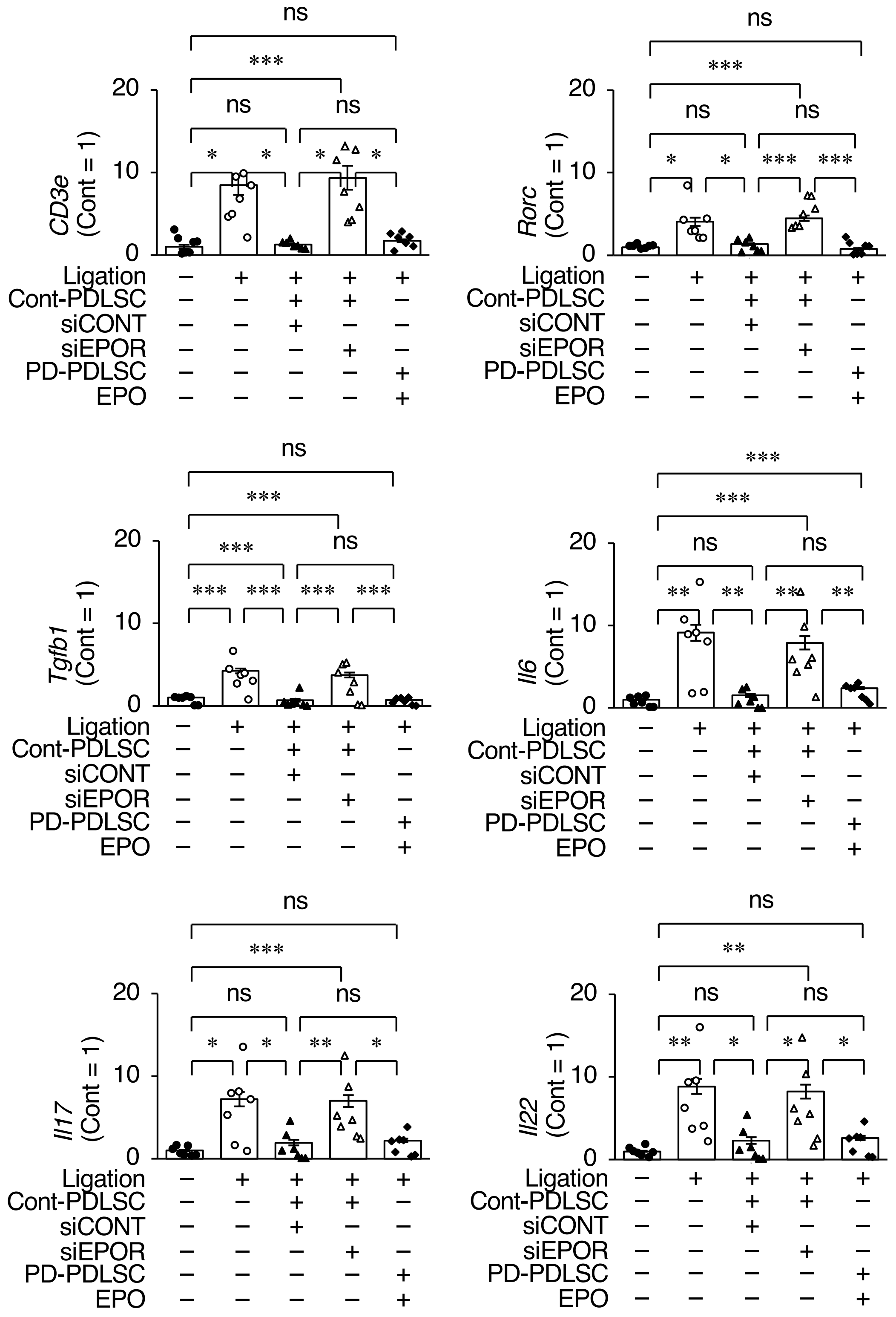

Fig. S5. EPOR signal modulates gingival immune condition in PDLSC-based therapy. Expression of *CD3e*, *retinoic acid receptor-related orphan receptor gamma* (*Rorc*), *transforming growth factor beta* (*Tgfb*), *interleukin 6* (*Il6*), *Il17*, *Il22* in gingiva around maxillary second molars by RT-qPCR. The results are shown as a ratio to the expression in control group (Cont = 1). Data are presented as mean ± SEM. n = 7/group. Significance was determined by two-way ANOVA with Tukey's post hoc test; ∗ $P < 0.05$, ∗∗ $P < 0.01$, and ∗∗∗ $P < 0.005$. ns, no significance.

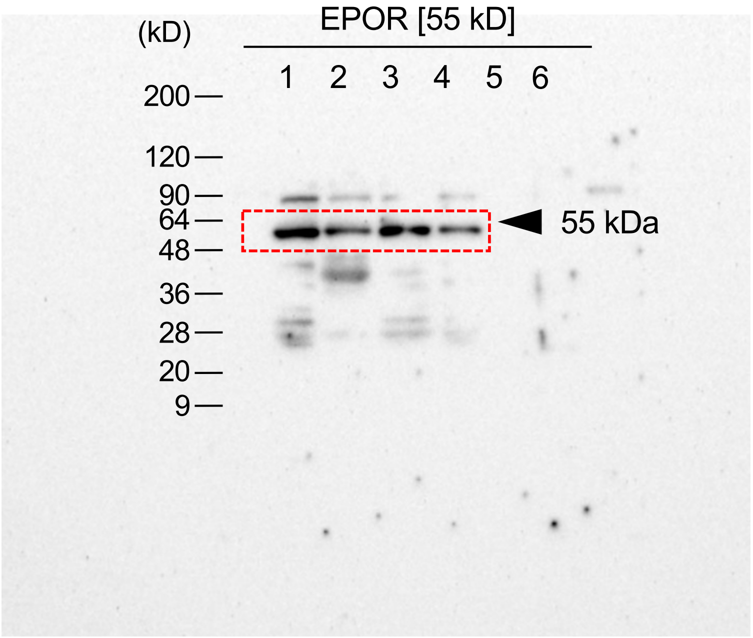

Lane 1: Cont-PDLSC
Lane 2: PD-PDLSC
Lane 3: siCONT-Cont-PDLSC
Lane 4: siEPOR-PDLSC
Lane 5: X
Lane 6: X

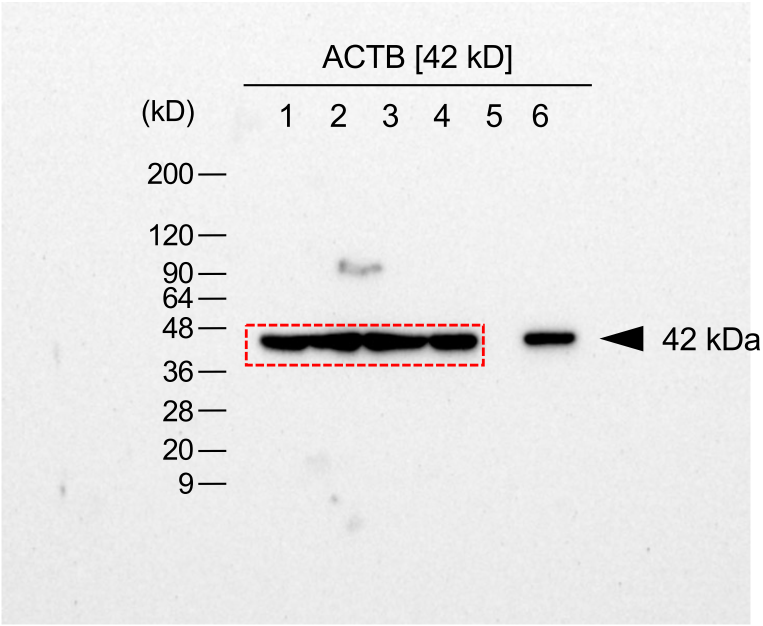

Lane 1: Cont-PDLSC
Lane 2: PD-PDLSC
Lane 3: siCONT-Cont-PDLSC
Lane 4: siEPOR-PDLSC
Lane 5: X
Lane 6: X

Figure S6. Full-length blot images from Fig 1c. The red-dot-lined squared area is used to indicate the corresponding cropped area. ACTB, actin beta; EPOR, erythropoietin receptor. Cont-PDLSC, healthy donor-derived periodontal ligament stem cells (PDLSCs); PD-PDLSC, periodontal disease patent-derived PDLSCs; siCONT-PDLSC, control-siRNA-treated PDLSCs; siEPOR-PDLSC, EPOR-siRNA-treated PDLSC.

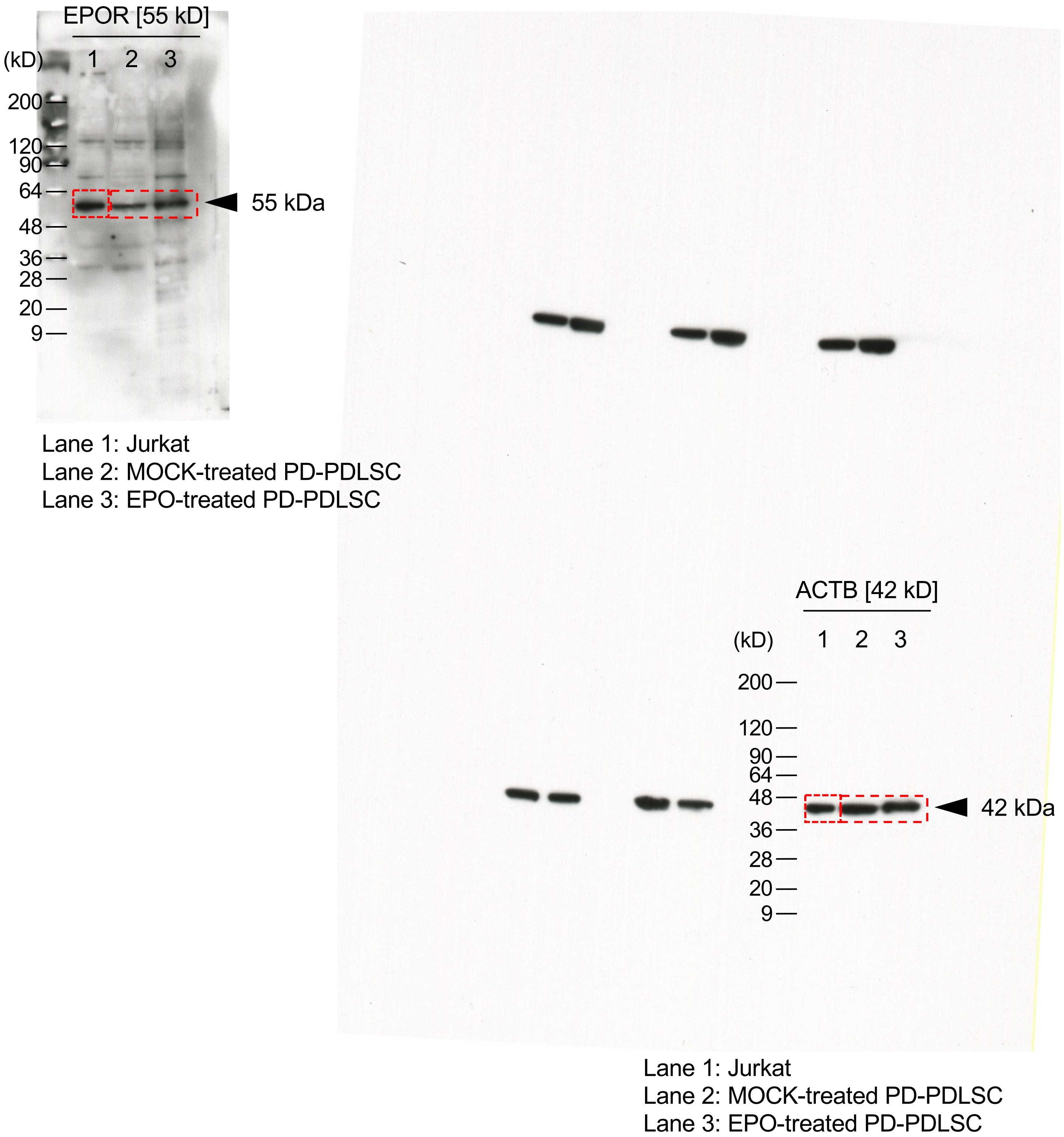

Figure S7. Full-length blot images from Fig 1c and f. The red-dot-lined squared area is used to indicate the corresponding cropped area. ACTB, actin beta; EPOR, erythropoietin receptor. PD-PDLSC, periodontal disease patent-derived PDLSCs; Jurkat, Jurkat cells; MOCK, PBS; EPO, erythropoietin.

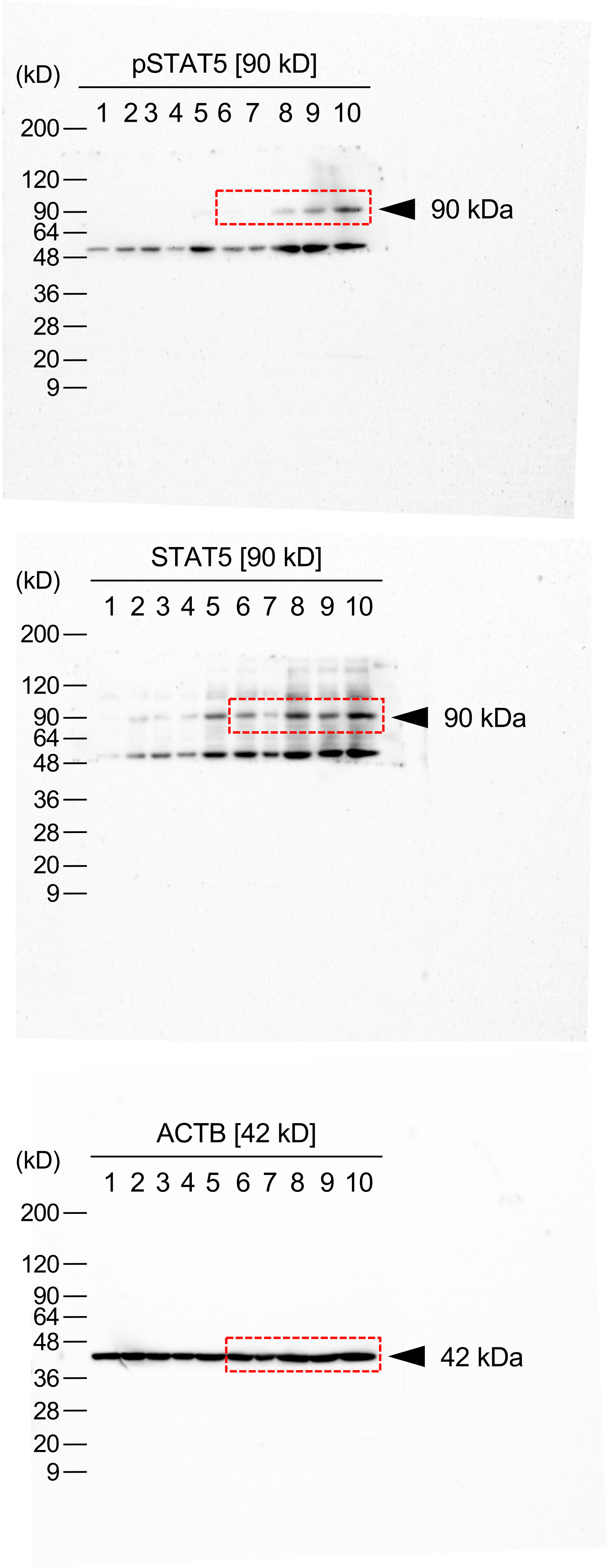

Figure S8. Full-length blot images from Fig 1g. The red-dot-lined squared area is used to indicate the corresponding cropped area. ACTB, actin beta; STAT5, signal transducer and activator of transcription 5. pSTAT5, phosphorylated STAT5.
