# Supplementary material for: Erythropoietin receptor signal is crucial for periodontal ligament stem cell-based tissue reconstruction in periodontal disease
Source: Sci Rep. 2024 Mar 20;14:6719. doi: 10.1038/s41598-024-57361-y (PMC10954634; doi:10.1038/s41598-024-57361-y)
Supplement: Supplementary file 2 — Supplementary Tables. [file 41598_2024_57361_MOESM2_ESM.pdf]

Supplementary Information

Erythropoietin receptor signal is crucial for periodontal ligament stem cell-based tissue reconstruction in periodontal disease

MDH. Fouad Zakaria, Soichiro Sonoda, Hiroki Kato, Lan Ma, Norihisa Uehara, Yukari Kyumoto-Nakamura, M. Majd Sharifa, Liting Yu, Lisha Dai, Erika Yamauchi-Tomoda, Reona Aijima, Haruyoshi Yamaza, Fusanori Nishimura, Takayoshi Yamaza

Supplementary Materials

Table S1. List of human- and muse-specific antibodies used for flow cytometry

Antibody, antigen	Antibody type, host, clone	Manufacturer
**Human specific antibody**		
anti-CD4 antibody, human	PerCP-conjugated IgG1 kappa, mouse, RPA-T4	BioLegend (San Diego, CA, USA)
anti-CD14 antibody, human	R-PE-conjugated IgG1 kappa, mouse, 63D3	BioLegend (San Diego, CA, USA)
anti-CD34 antibody, human	R-PE-conjugated IgG2a kappa, mouse, 561	BioLegend (San Diego, CA, USA)
anti-CD45 antibody, human	R-PE-conjugated IgG1 kappa, mouse, 2D1	BioLegend (San Diego, CA, USA)
anti-CD73 antibody, human	R-PE-conjugated IgG1 kappa, mouse, AD2	BioLegend (San Diego, CA, USA)
anti-CD105 antibody, human	R-PE-conjugated IgG1 kappa, mouse, 43A3	BioLegend (San Diego, CA, USA)
anti-CD146 antibody, human	R-PE-conjugated IgG1 kappa, mouse, P1H112	BioLegend (San Diego, CA, USA)
anti-EPOR antibody, human	R-PE -conjugated IgG1 kappa, mouse, 38421	R&D Systems (Minneapolis, MN, USA
anti-IFNG antibody, human	APC-conjugated IgG1 kappa, mouse, 4S.B3	BioLegend (San Diego, CA, USA)
anti-IL17 antibody, human	R-PE-conjugated IgG1 kappa, mouse, 9D3.1C8	BioLegend (San Diego, CA, USA)
control mouse IgG1 kappa	R-PE-conjugated IgG1 kappa, mouse, MOPC-21	BioLegend (San Diego, CA, USA)
control mouse IgG2a kappa	R-PE-conjugated IgG2a kappa, mouse, MOPC-173	BioLegend (San Diego, CA, USA)
**Mouse specific antibody**		
**Antibody, antigen**	**Antibody type, host, clone**	**Manufacturer**
anti-CD11b antibody, mouse	APC-conjugated IgG2a kappa, rat, M1/70	BioLegend (San Diego, CA, USA)
anti-CD45.2 antibody, mouse	Alexa Flour488-conjugated IgG2a kappa, rat, 104	BioLegend (San Diego, CA, USA)
anti-Ly6G antibody, mouse	R-PE-conjugated IgG2a kappa, rat, 1A8	BioLegend (San Diego, CA, USA)
control rat IgG2a kappa	R-PE-conjugated IgG2a kappa, rat, RTK2758	BioLegend (San Diego, CA, USA)

EPOR, erythropoietin receptor; IFNG, interferon gamma; IL17, interleukin 17; Ly6G, lymphocyte antigen 6 complex locus. APC, allophycocyanin; R-PE, R-phycoerythrin.

Table S2. List of specific antibodies used for immunoblotting, immunofluorescence, and immunohistochemistry.

Antibody, antigen	Antibody type, host, clone	Manufacturer
anti-ACTB antibody, mouse	purified IgG1, mouse, AC-15	Millipore Sigma (Burlington, MA, USA)
anti-EPOR antibody, human	purified IgG1 kappa, mouse, 38421	R&D Systems (Minneapolis, MN, USA
anti-human mitochondria antibody, human	purified, mouse IgG1, 113-1	Abcam (Cambridge, UK)
anti-STAT5 antibody, human	purified IgG, rabbit	Cell Signaling Technology (Danverse, MA, USA)
anti-PhosphoSTAT5 antibody, human	purified IgG, rabbit	Cell Signaling Technology (Danverse, MA, USA)
control mouse IgG1 kappa	purified IgG1 kappa, mouse, MOPC-21	BioLegend (San Diego, CA, USA)

ACTB, actin, beta; EPOR, erythropoietin receptor; STAT5, signal transducer and activator of transcription 5; PhosphoSTAT5, phosphorylated STAT5.

Table S3. List of TaqMan probes used for human and mouse gene analysis.

Human gene	Gene assay ID	Manufacturer
ACAN	Hs00153936_m1	Thermo Fisher Scientific (Waltham, MA, USA)
BGLAP	Hs01587814_g1	Thermo Fisher Scientific (Waltham, MA, USA)
COL1A1	Hs00164004_m1	Thermo Fisher Scientific (Waltham, MA, USA)
EPOR	Hs00959427_m1	Thermo Fisher Scientific (Waltham, MA, USA)
LPL	Hs00173425_m1	Thermo Fisher Scientific (Waltham, MA, USA)
POSTN	Hs01566750_m1	Thermo Fisher Scientific (Waltham, MA, USA)
PPARG	Hs0115513_m1	Thermo Fisher Scientific (Waltham, MA, USA)
RUNX2	Hs00231692_m1	Thermo Fisher Scientific (Waltham, MA, USA)
SCX	Hs03054636_g1	Thermo Fisher Scientific (Waltham, MA, USA)
SEMA3A	Hs00173810_m1	Thermo Fisher Scientific (Waltham, MA, USA)
SOX9	Hs01001343_g1	Thermo Fisher Scientific (Waltham, MA, USA)
TERT	Hs00972650_m1	Thermo Fisher Scientific (Waltham, MA, USA)
18S rRNA	Hs99999901_s1	Thermo Fisher Scientific (Waltham, MA, USA)
**Mouse gene**	**Gene assay ID**	**Manufacturer**
CD3e	Mm01179194_m1	Thermo Fisher Scientific (Waltham, MA, USA)
Il1b	Mm00434228_m1	Thermo Fisher Scientific (Waltham, MA, USA)
Il6	Mm00446190_m1	Thermo Fisher Scientific (Waltham, MA, USA)
Il17	Mm00439618_m1	Thermo Fisher Scientific (Waltham, MA, USA)
Il22	Mm01226772_g1	Thermo Fisher Scientific (Waltham, MA, USA)
Ly6g	Mm04934123_m1	Thermo Fisher Scientific (Waltham, MA, USA)
Rank	Mm00437135_m1	Thermo Fisher Scientific (Waltham, MA, USA)
Rorc	Mm01261022_m1	Thermo Fisher Scientific (Waltham, MA, USA)
Tgfb1	Mm00436955_m1	Thermo Fisher Scientific (Waltham, MA, USA)
Tnfa	Mm00443258_m1	Thermo Fisher Scientific (Waltham, MA, USA)
18S rRNA	Mm03928990_g1	Thermo Fisher Scientific (Waltham, MA, USA)

ACAN, aggrecan; BGLAP, bone gamma-carboxyglutamate protein; COL1A1, collagen, type I, alpha 1; EPOR, erythropoietin receptor; LPL, lipoprotein lipase; POSTN, periostin; PPARG, peroxisome proliferator-activated receptor gamma; RUNX2, runt-related transcription factor 2; SCX, scleraxis BHLH transcription factor; SEMA3A, semaphoring 3A SOX9, SRY-box9; TERT, telomerase reverse transcriptase. CD3e, CD3 epsilon; Il1b, interleukin 1 beta; Il6, interleukin 6; Il17, interleukin 17; Il22, interleukin 22; Ly6g, lymphocyte antigen 6 complex locus G; Rank, receptor activator of NF-κB; Rorc, retinoic acid receptor-related orphan receptor gamma; Tgfb1, transforming growth factor beta 1; Tnfa, tumor necrosis factor alpha.

Table S4. Clinical dental information of donors

Donor#	Age (years)	Sex	Donor tooth	
			Position	Tooth condition
Cont#1	19	Female	48	Impaction
Cont#2	23	Female	48	Impaction
Cont#3	20	Male	38	Impaction
PD#1	52	Female	46	BOP, PPD = 9 mm, TM III
PD#2	65	Male	25	BOP, PPD = 7 mm, TM III
PD#3	43	Female	27	BOP, PPD = 8 mm, TM III

A tooth with irreversibly severe chronic periodontitis and an impacted third molar with periodontally healthy were selected as periodontal disease (PD) and healthy control (Cont) donor samples, respectively. Severe periodontitis were diagnosed by the following symptoms including bleeding upon probing (BOP) as a clinical symptom of gingival inflammation, more than one periodontal pocket depth (PPD) of >5 mm, and clinical degree III of tooth mobility (TM).
